# The biology and treatment of Merkel cell carcinoma: current understanding and research priorities

**DOI:** 10.1038/s41571-018-0103-2

**Published:** 2018-10-04

**Authors:** Paul W. Harms, Kelly L. Harms, Patrick S. Moore, James A. DeCaprio, Paul Nghiem, Michael K. K. Wong, Isaac Brownell, on behalf of the International Workshop on Merkel Cell Carcinoma Research (IWMCC) Working Group

**Affiliations:** 1 Department of Pathology, University of Michigan Medical School, Ann Arbor, MI USA; 2 Department of Dermatology, University of Michigan Medical School, Ann Arbor, MI USA; 3 Cancer Virology Program, University of Pittsburgh, Pittsburgh, PA USA; 4 Department of Medical Oncology, Dana-Farber Cancer Institute, Boston, MA USA; 5 Department of Medicine, Division of Dermatology, University of Washington, Seattle, WA USA; 6 Department of Melanoma Medical Oncology, Division of Cancer Medicine, MD Anderson Cancer Center, Houston, TX USA; 7 Dermatology Branch, National Institute of Arthritis and Musculoskeletal and Skin Diseases (NIAMS) and National Cancer Institute (NCI), NIH, Bethesda, MD USA

**Keywords:** Neuroendocrine cancer, Skin cancer, Cancer immunotherapy, Targeted therapies

## Abstract

Merkel cell carcinoma (MCC) is a rare and aggressive skin cancer associated with advanced age and immunosuppression. Over the past decade, an association has been discovered between MCC and either integration of the Merkel cell polyomavirus, which likely drives tumorigenesis, or somatic mutations owing to ultraviolet-induced DNA damage. Both virus-positive and virus-negative MCCs are immunogenic, and inhibition of the programmed cell death protein 1 (PD-1)–programmed cell death 1 ligand 1 (PD-L1) immune checkpoint has proved to be highly effective in treating patients with metastatic MCC; however, not all patients have a durable response to immunotherapy. Despite these rapid advances in the understanding and management of patients with MCC, many basic, translational and clinical research questions remain unanswered. In March 2018, an International Workshop on Merkel Cell Carcinoma Research was held at the US National Cancer Institute, at which academic, government and industry experts met to identify the highest-priority research questions. Here, we review the biology and treatment of MCC and report the consensus-based recommendations agreed upon during the workshop.

## Abstract (web summary)

Merkel cell carcinoma (MCC) is a rare and aggressive form of nonmelanoma skin cancer. The availability of immune checkpoint inhibition has improved the outcomes of a subset of patients with MCC, although many unmet needs continue to exist. In this Consensus Statement, the authors summarize developments in our understanding of MCC while also providing consensus recommendations for future research.

## Introduction

**Fig. 1:**
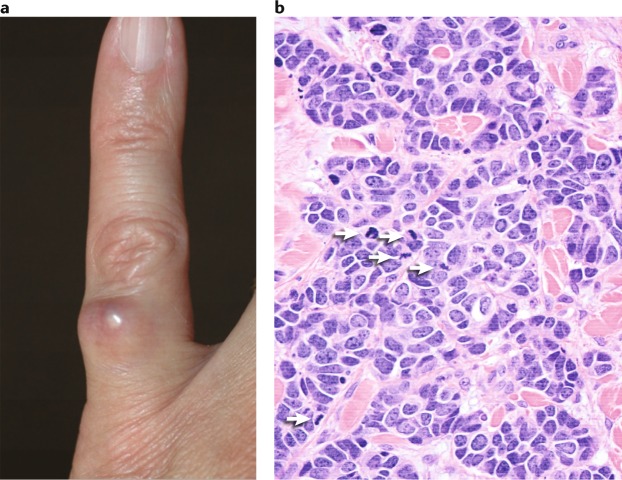
Clinical and histological appearance of MCC. **a** | Photograph depicting the clinical appearance of Merkel cell carcinoma (MCC), presenting as a rapidly growing nodule on an extremity; such lesions can also be commonly observed on a patient’s head or neck. **b** | Light micrograph (×400 magnification) of a sample stained with haematoxylin and eosin depicting the histological appearance of MCC, demonstrating the presence of round cells with scant cytoplasm, neuroendocrine chromatin and numerous mitotic figures (arrows). Trabecular patterning might also be prominent.

Our understanding of the biology of Merkel cell carcinoma (MCC) and the management of patients with this disease has advanced exponentially since the first description of this disease in 1972 (ref.^1^). MCC, also known as primary cutaneous neuroendocrine carcinoma, is named owing to its ultrastructural and immunophenotypic resemblance to sensory Merkel cells found in the skin (Fig. 1). MCC is frequently metastatic and has an estimated 33–46% disease-specific mortality^2,3^. In most parts of the world, the majority of MCCs are caused by the monoclonal integration of Merkel cell polyomavirus (MCPyV)^4^, and the remainder are associated with exposure to ultraviolet light^5–7^. Therapeutic options for patients with advanced-stage MCC have historically been limited; however, new immunotherapeutic approaches are enabling durable responses to be achieved in a subset of patients^8^. In this Consensus Statement, we review the current state of knowledge of MCC biology and treatment and define key outstanding questions in the areas of basic, translational and clinical MCC research.

## Biological features of MCC

### Merkel cell polyomavirus

MCPyV is a common virus and the causative agent in most MCCs. An association between MCPyV and MCC was first identified using digital transcriptome subtraction (filtering out known human RNA sequences in order to identify potential viral transcripts)^4^. The Polyomaviridae family of small double-stranded DNA viruses, to which MCPyV belongs, includes other polyomaviruses associated with cutaneous infection in humans (*Trichodysplasia spinulosa* polyomavirus, human polyomavirus 6 and human polyomavirus 7) or diseases of other organ systems (JC polyomavirus, BK polyomavirus, WU polyomavirus and KI polyomavirus)^9^. To date, MCPyV is the only known human oncovirus in the polyomavirus family; why MCPyV holds this distinctive status is currently unknown.

The prevalence of subclinical MCPyV infection increases with age, with a seroprevalence in adults of approximately 60–80%^10–18^. The skin is a major site of viral infection, although MCPyV has also been detected in peripheral blood and a range of other organ systems^10,19–26^. MCPyV infection seems to be asymptomatic^11^.

The specific host cell type for MCPyV infection has thus far remained elusive. Benign Merkel cells are not sufficiently numerous to account for the MCPyV burden typically detected in skin^27^. Peripheral blood monocytes have been proposed to act as a reservoir of infected cells^24^. MCPyV reporter pseudovirus can enter many cell types, including keratinocytes^28,29^, although dermal fibroblasts and HEK 293 cells are the only cells in which productive viral infection has been demonstrated in vitro^28,30^.

**Fig. 2:**
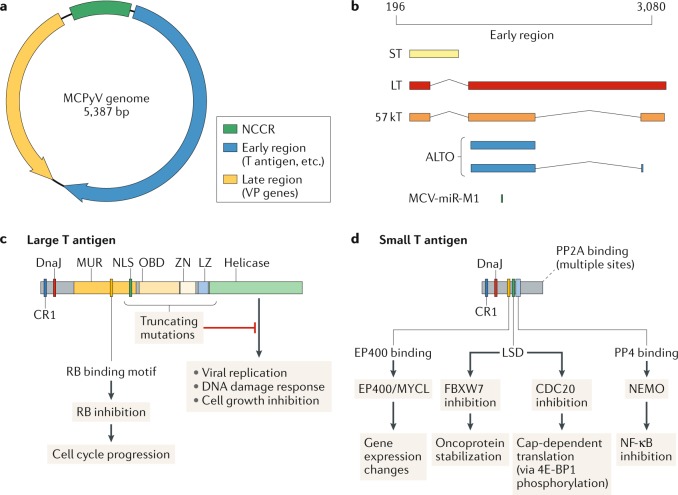
Structure and function of the Merkel cell polyomavirus genome. **a** | Merkel cell polyomavirus (MCPyV) is a small double-stranded DNA virus with a 5,387 bp circular genome that includes a non-coding control region (NCCR), an early region containing T antigen genes that coordinate viral replication and a late region containing viral protein (VP) genes for virion capsid proteins. **b** | Multiple transcripts are generated from the early region by alternative splicing and possibly alternative start sites, including large T antigen (LT), small T antigen (ST), 57 kT antigen (57 kT), alternative frame of the large T open reading frame (ALTO) and microRNA MCV-miR-M1. **c** | Cellular functions of the MCPyV LT. The DnaJ domain mediates binding to heat shock cognate 71 kDa protein, with a role in viral replication. The MCPyV unique region (MUR) includes the retinoblastoma-associated protein (RB) binding motif responsible for direct inhibition of RB, thus enabling cell cycle progression to S phase. The MUR mediates binding to the vacuolar sorting protein VPS39. The carboxyl-terminal helicase domain is a critical mediator of viral replication, with contributions from the adjacent zinc-finger (ZN), leucine zipper (LZ) and origin binding domain (OBD). The integrated MCPyV genome present in MCPyV-positive MCCs harbours truncating mutations in LT that disrupt the helicase domain, resulting in a replication-incompetent mutant form of LT that nonetheless retains the ability to promote cell cycle progression. **d** | Cellular functions of MCPyV ST. ST recruits L-MYC to the EP400 chromatin remodelling complex in order to mediate changes in gene expression. The LT-stabilizing domain (LSD) is proposed to inhibit E3 ubiquitin ligase activity via interactions with F-box/WD repeat-containing protein 7 (FBXW7) and cell division cycle protein 20 homologue (CDC20), resulting in increased oncoprotein stability and cap-dependent mRNA translation, respectively. ST also interacts with the protein phosphatase complex PP4 to inhibit nuclear factor-κB (NF-κB) signalling. 4E-BP1, eukaryotic translation initiation factor 4E-binding protein 1; CR1, conserved region 1; NEMO, NF-κB essential modulator; NLS, nuclear localization signal.

The viral life cycle of MCPyV is similar to that of other polyomaviruses. The episomal viral genome possesses an early region (ER) and late region (LR), which contain genes encoding proteins that coordinate viral replication and viral capsid proteins, respectively^31^ (Fig. 2). Genes in the ER of MCPyV encode the large T antigen (LT), small T antigen (ST) and 57 kT antigen transcripts (Fig. 2b). The middle T-like overprinting gene large T open reading frame (*ALTO*) is also proposed to be located in the ER^32–34^. The MCPyV LR encodes the viral protein 1 (VP1) and VP2 capsid proteins^31,35^ and might enable expression of the microRNA MCV-miR-M1-5p (which is located on the LR strand despite mapping among genes in the ER)^33,34,36^. Productive viral infection is associated with host cell death rather than oncogenic transformation^30^. MCPyV replication is regulated by E3 ligase targeting of phosphorylated LT, as well as feedback inhibition by LT on its own promoter^30^ and inhibition of LT production by viral microRNA^36^. These features act together to inhibit viral replication after entry into a host cell; the virus typically then defaults to a latent, nonreplicative state after infection^30,34^.

**Fig. 3:**
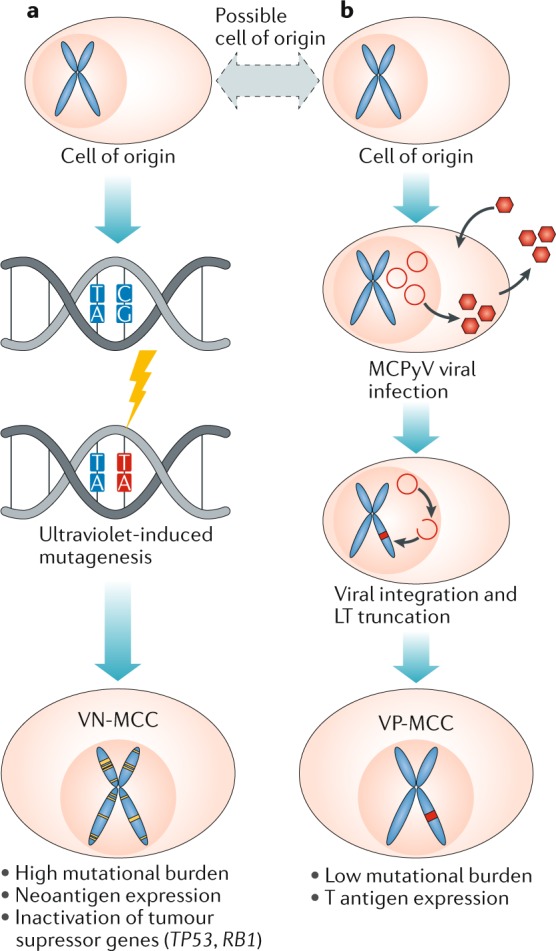
Proposed MCC tumorigenesis pathways in the presence or absence of Merkel cell polyomavirus. **a** | In virus-negative Merkel cell carcinomas (VN-MCCs), the cell of origin undergoes ultraviolet-mediated DNA damage, resulting in a high tumour mutational burden and inactivation of tumour suppressor genes, including *RB1* and *TP53*. The high mutational burden might result in the expression of tumour neoantigens that represent potential targets for antitumour immunity. **b** | In virus-positive MCC (VP-MCC), the cell of origin is infected by wild-type Merkel cell polyomavirus (MCPyV), which undergoes episomal replication. Rarely, MCPyV can become integrated into the host cell genome and further acquires a truncating mutation of the large T antigen (LT), resulting in deficient viral replication with continued production of viral oncoproteins. The resulting tumour has a low burden of cellular genomic mutations. Hence, for patients with VP-MCCs, T antigen proteins (rather than neoantigens) might be better targets for treatments designed to promote antitumour immunity.

Oncogenic transformation by MCPyV is hypothesized to require two events: integration of the viral genome into the host genome and truncation of LT to render the viral genome incapable of replication^4,37^ (Fig. 3). Viral integration into the host genome might occur by accidental genome fragmentation during MCPyV replication; the precise location of the integration site seems to be random, without consistent involvement of specific cellular tumour suppressor genes or oncogenes^4,38–42^. In virus-positive (VP)-MCC, mutations in *MCPyV gp3* encoding LT disrupt the DNA binding domain and the helicase domain distal to the retinoblastoma-associated protein (RB) binding motif (Fig. 2c). The resulting truncated LT retains its ability to bind to RB and promote cell cycle progression^37^ but cannot mediate viral replication^43,44^. The integrated and mutated virome no longer produces MCPyV virions. The very low probability of this required combination of events might explain why MCC is rare despite the apparent ubiquity of MCPyV infection.

LT and ST have diverse activities that might contribute to oncogenesis. Evidence suggests that cultured VP-MCC cells are dependent upon the presence of both viral T antigens for proliferation and survival^45–47^. The common amino-terminal region of viral T antigens associates with heat shock cognate 71 kDa protein, with roles in the regulation of viral replication and possibly tumour cell proliferation^43,48^ (Fig. 2c,d). Similar to the SV40 polyomavirus large T antigen, the MCPyV LT directly binds with and inactivates the RB (Fig. 2c). This interaction has been shown to enhance expression of the oncoprotein survivin, which might be an important therapeutic target in patients with VP-MCC tumours^49^. Unlike the SV40 large T antigen, MCPyV LT has not been shown to directly bind with p53 (ref.^50^). MCPyV LT is also able to associate with the vacuolar sorting protein Vam6/Vps39-like protein, which has potential importance for viral replication^51^. Additional functions of LT, which are likely to be lost after truncation, include DNA binding, bromodomain protein 4 (BRD4) binding, helicase activity and inhibition of cell growth^31,52^. The tumour-associated mutations that truncate LT in MCPyV are also likely to disrupt *ALTO*^32^.

Multiple lines of evidence suggest that ST has a critical role in VP-MCC oncogenesis. ST expression is sufficient to transform rat-1 fibroblasts in culture^53^. Data from transgenic mouse models indicate that ST expression is transformative in various organ systems, including in the epidermis^54–57^. In mouse models, MCPyV T antigens alone are generally not sufficient to induce the formation of neuroendocrine tumours^55^; however, co-expression of ST with the transcription factor ATOH1 generates intraepidermal MCC-like proliferations^57^ and, with the loss of *TP53*, undifferentiated anaplastic tumours^54^.

ST has a diverse range of cellular activities (Fig. 2d). Similar to other small T antigens, the MCPyV ST has a PP2A region that binds with and inhibits various components of the serine/threonine phosphatase complexes PP2A and PP4 (ref.^58^). Interactions between ST and PP4 might also mediate inhibition of the nuclear factor-κB (NF-κB) pathway, as well as promote cell motility via effects on the actin and microtubular cytoskeleton^59–62^. The PP2A binding domain is not required for cellular transformation by MCPyV ST^31^, indicating that other domains of ST are crucial for oncogenesis.

The MCPyV ST has a distinct domain known as the LT-stabilizing domain (LSD) (Fig. 2d), which has been proposed to inhibit the activity of several E3 ubiquitin ligases^63,64^. E3 ubiquitin ligases regulate cell cycle progression and other processes by targeting specific proteins for degradation. ST might bind with and inhibit the F-box/WD repeat-containing protein 7 (FBXW7) component of the SKP1–CUL1–F-box protein (SCF)–E3 ubiquitin ligase complex, resulting in decreased degradation of various oncoproteins^63^. Co-expression of ST and LT leads to stabilization of LT, which might be mediated via inhibition of FBXW7 by ST to prevent the E3 ubiquitin ligase-mediated degradation of LT (although neither protein harbours the classic high-affinity phosphodegrons required for FBXW7 binding). Other oncogenic proteins, including MYC, might be stabilized by the ST via a similar mechanism^63^. Interaction of the LSD domain with cell division cycle protein 20 homologue (CDC20), an activator of the anaphase-promoting complex (also known as the cyclosome) ubiquitin ligase, has been reported to trigger a series of signalling events culminating in the inhibitory phosphorylation of the eukaryotic translation initiation factor 4E-binding protein 1 (4E-BP1)^64^, leading to derepression of cap-dependent mRNA translation with potential pro-survival effects^65^. The LSD domain is required for oncogenic activity by ST in vitro and in vivo, thus supporting a critical role of this domain in the oncogenesis of MCPyV-induced MCC^55,57,63^.

ST has been shown to bind with L-MYC to regulate the EP400 histone acetyltransferase and chromatin remodelling complex^66^ (Fig. 2d). Additional activities involving ST include increased aerobic glycolysis, possibly via the MYC and/or NF-κΒ signalling pathways^67^.

### Detection of tumour-associated MCPyV

Methods used for the experimental detection of MCPyV in tumours include immunohistochemistry, PCR, RNA or DNA in situ hybridization and next-generation sequencing (NGS)^39,68–72^. These assays vary substantially in terms of sensitivity and specificity for the detection of tumour-associated MCPyV. Measuring the expression of T antigen proteins determined using immunohistochemistry is a common approach for the detection of MCPyV. The most broadly used antibody for MCPyV LT detection, CM2B4, is commercially available and has an approximately 88% sensitivity and 94% specificity (compared with that of multimodal approaches combining PCR with immunohistochemistry)^73^. Nonspecific staining with CM2B4 can be observed in both tonsillar and lymphoid tissues^73,74^. Other antibodies recognizing the LT, ST or the common T antigen region have been reported and might also be useful in detecting viral proteins^73^.

PCR is another common method of MCPyV detection. The 5ʹ end of the second exon of LT is frequently targeted using this approach^70^. Compared with multimodal MCPyV detection, PCR-based amplification within the second exon of LT had 83% sensitivity and 81% specificity^73^. The sensitivity of PCR-based MCPyV detection can be improved by additional PCR assays targeting other amplicons in the ER, including *MCPyV gp4* encoding ST^70^. Quantitative PCR (qPCR) enables estimation of the number of integrated MCPyV copies per host cell genome. Copy numbers can be quantified by comparisons to the reference MCC cell line MKL-2, which has the lowest relative MCPyV copy number among established VP-MCC cell lines and is thus estimated to have a single viral copy per cell^70^. Similar results are obtained by comparing MCPyV qPCR values to those of selected reference genes from the human genome^71,75,76^. MCPyV copy number estimates can range from extremely low (<1 copy per 100 cells) to thousands of copies per cell^70^. The reasons for extremely low MCPyV copy numbers are incompletely understood but might be caused by technical factors, including inefficient PCR amplification owing to mutations in the integrated MCPyV genome, low purity of tumour samples or the detection of infectious wild-type MCPyV in the adjacent nonmalignant skin^77^. In low-purity tumour samples, copy number estimates of tumour-associated MCPyV might be confounded by background signals from skin and/or non-MCC tumours^69,77^. qPCR does not allow for visual confirmation that positive results are associated with tumour cells; therefore, background MCPyV infection cannot be excluded in MCC tumours with low signal^69^. A multimodal approach incorporating both PCR and immunohistochemistry is likely to be a more sensitive and specific method for confirming MCPyV status among commonly used assays^73^.

Other newer approaches for MCPyV detection have been less extensively investigated but might have advantages over immunohistochemistry and/or PCR. RNA in situ hybridization might provide similar levels of sensitivity to that of PCR while also enabling visual correlation with tissue morphology and the exclusion of background infection^69^. NGS can be effective in detecting MCPyV sequences, including tumour-specific truncating mutations. NGS using a hybrid capture approach can further demonstrate the presence of viral integration sites^39^ and therefore provides the greatest level of specificity for confirming the presence of tumour-specific MCPyV. However, the time, expense and expertise required for hybrid capture NGS currently make this approach impractical for many diagnostic and research laboratories.

### Non-viral changes in MCC

Similar to other malignancies, MCCs typically harbour genomic aberrations including chromosomal copy number variations (CNVs) and other mutations. However, significant differences in the patterns of these various changes exist between VP-MCCs and virus-negative (VN)-MCCs. A standard terminology for designating the viral status of MCCs has not been established. We propose the terms VP-MCC and VN-MCC to describe virus-positive and virus-negative MCCs, respectively, as useful standard abbreviations for this purpose.

Most MCCs harbour chromosomal CNVs, regardless of viral status^78^. VP-MCCs typically have fewer CNVs than VN-MCCs^78^. MCCs are generally heterogeneous with regard to patterns of chromosomal alterations^78,79^. However, certain alterations are recurrent in a minority of tumours. Gains of chromosome 1p34, including the *MYCL* oncogene, occur in approximately 39% of tumours and can be observed in both VP-MCCs and VN-MCCs^78^. Deletions affecting *RB1* are also frequent (approximately 26% of tumours) and also occur in both VP-MCCs and VN-MCCs^78,80,81^.

VP-MCCs and VN-MCCs have distinct patterns of somatic mutation. VN-MCCs have a high mutational burden, an ultraviolet mutational signature and highly recurrent inactivation of tumour suppressor genes, including *TP53*, *RB1* and genes encoding members of the Notch family of signalling proteins^5–7,82^ (Fig. 3). By contrast, VP-MCCs tend to have a low mutational burden, no definitive mutational signature and an absence of *TP53* and *RB1* mutations^5–7^. Mutations in VP-MCCs are predominantly subclonal, suggesting that the majority of these mutations occur late in tumour evolution rather than as early or founder events^79^, possibly arising owing to transcription-coupled damage or in the setting of double-stranded DNA breaks^38^. By contrast, mutations in *TP53* and *RB1* in VN-MCCs are typically clonal and shared by primary tumours and their matched metastases^79^. Hotspot activating mutations of several different oncogenes are observed in both VP-MCCs and VN-MCCs, including *HRAS*, *KRAS* and *PIK3CA*^5–7,83–85^. Other hotspot activating mutations have been described in VN-MCCs, but not in VP-MCCs, including mutations in *KNSTRN*, *RAC1*, *AKT1*, *CTNNB1* and *EZH2* (refs^5,7,83,86^). Most VN-MCCs have detectable activation of at least one known proto-oncogene.

The genomic changes observed in MCC have implications for the relative contributions of the major intracellular signalling cascades to tumorigenesis. A minority of MCCs harbour mutations or chromosomal copy number alterations predicted to result in activation of the AKT–PI3K signalling pathway, affecting genes including *PIK3CA*, *PIK3CG*, *AKT, PTEN, PREX2* and *TSC1* (refs^5–7,83,85,87^). The MAPK signalling pathway is not constitutively activated in MCCs^88^, and reports have been mixed regarding whether MAPK activation promotes proliferation or apoptosis of cultured MCC cells in vitro^89,90^. *HRAS* hotspot mutations in MCC have been associated with increased ERK phosphorylation but not with sensitivity to MEK inhibitors in an analysis involving VN-MCC cell lines^7^; the possibility that RAS might also activate other pathways, including the PI3K pathway^91^, has not been investigated in this context. Coding mutations in receptor tyrosine kinases and RAF family members can also occur in MCC, although classic activating mutations or fusions affecting the genes encoding these proteins have yet to be identified^5–7,86,88,92–95^. Evidence suggests that activation of Wnt signalling is not a common event in MCC tumours^96–98^, although activating β-catenin (*CTNNB1*) mutations^86^ or nuclear β-catenin accumulation^96,97^ can both be detected in small subsets of tumours. Molecular studies have failed to detect activation of Hedgehog signalling in most MCCs^99^, despite histological detection of Hedgehog pathway proteins^100,101^. Functional studies of these and other oncogenic pathways in VN-MCCs have been confounded by a lack of transgenic mouse models^102^ and the atypical biology of several MCPyV-negative cell lines^103,104^.

Epigenetic deregulation, including modifications of both DNA (especially promoter silencing by CpG island hypermethylation) and histones, can also contribute to tumour aggressiveness. Promoter hypermethylation in MCC can affect several genes, including *DUSP2*, *CDKN2A* and members of the *RASSF* family^105–108^. The polycomb group complex, which includes the histone-lysine *N*-methyltransferase EZH2, mediates gene silencing via histone H3 lysine 27 trimethylation and can also be deregulated in MCC^83,109,110^. Notably, epigenetic silencing of HLA genes by histone deacetylases might contribute to immune evasion in the development of MCC^111–113^.

### MCC cell of origin

Identifying the cell type from which a neoplasm arises has implications for ascertaining the mechanisms of tumour initiation, experimental modelling and possibly uncovering therapeutic susceptibilities. MCC is a poorly differentiated neuroendocrine carcinoma that lacks a recognized benign or dysplastic precursor. Furthermore, MCCs are most frequently found in the dermis but can arise from any layer of the skin (from intraepidermal to subcutaneous)^3^. Thus, fundamental evidence on the MCC cell of origin is currently lacking. MCC cells have immunophenotypic and ultrastructural similarities to benign Merkel cells; however, Merkel cells are postmitotic in vivo, and the anatomical regions with the highest Merkel cell density are not the most frequent sites of MCC detection^3,31^. On the basis of these observations, most investigators do not consider mature Merkel cells to be a likely candidate for the MCC cell of origin^9^. Instead, MCCs are more likely to be derived from a population that recapitulates the Merkel cell differentiation pathway either before or during neoplastic transformation. In addition, given that VN-MCCs have ultraviolet mutational signatures whereas VP-MCCs do not, these tumour types might arise from distinct cells of origin with different levels of baseline DNA photodamage^102^.

Proposed candidates for the MCC cell of origin include pro-B cells or pre-B cells, fibroblasts, dermal mesenchymal stem cells and epidermal progenitor cell populations, among others^28,55,57,114–116^. Currently, no consensus has been reached regarding the most likely candidate for the MCC cell of origin. A comprehensive discussion of the evidence related to each putative cell of origin is beyond the scope of this article.

Other virus-associated malignancies might be informative when considering candidates for the MCC cell of origin. In cervical squamous cell carcinomas, the activity of viral oncogenes in intraepithelial precursor lesions that progress to epithelial tumours supports human papilloma virus (HPV)-infected epithelial stem cells as the cells of origin^117^. For patients with Kaposi sarcoma herpesvirus (KSHV)-associated primary effusion lymphoma, recent observations have raised the possibility that mesothelial cells might be the cell of origin via mesenchymal-to-lymphoid transformation^118^; therefore, tumour phenotype might not always be a reliable predictor of the cell of origin.

## Clinical features of MCC

### Epidemiology

As of 2013, the annual incidence of MCC in the USA was 0.7 cases per 100,000 people^119^. The incidence of MCC in the USA almost doubled between 2000 and 2013 and is expected to exceed 3,000 cases per year by 2025 (ref.^119^), with similar increases in Australia and many but not all European countries^120^. The basis for this increasing incidence is unclear but might be related to an ageing population and improvements in diagnostic recognition^119^. The frequency of MCC is higher closer to the equator and much lower among those of non-white ethnicities^120,121^, suggesting an association with sensitivity to ultraviolet radiation. Australia currently has the highest reported incidence of MCC (up to 1.6 cases per 100,000 person-years)^121^, notably with a higher percentage of VN-MCCs, probably reflecting a higher risk of environmental ultraviolet exposure^122–124^. MCC also has a higher incidence among immunosuppressed populations^125^. Apart from immunosuppressed individuals, MCC arises almost exclusively in those of an advanced age. Incidence estimates fall below zero in patients under age <40 years of age^119^, and 90% of patients are >50 years of age^126^. Unlike many other cancers, in which disease incidence peaks and then declines with increasing age, the incidence of MCC continues to increase even as patients reach 80 or even 90 years of age, possibly owing to immune senescence^119^. Thus, MCC affects populations that frequently have substantial comorbidities that could complicate patient management.

### Presentation and diagnosis

MCC classically presents as a rapidly growing red or violaceous nodule on the sun-exposed skin of an elderly, fair-skinned individual. However, up to 15% of patients with MCC will present with a tumour-positive lymph node without an identifiable cutaneous tumour, presumably reflecting metastatic disease with regression of the primary skin tumour^3,9,127^.

Confirming a diagnosis of MCC requires evaluation of histopathological and immunohistochemical findings. The histopathological differential diagnosis might include lymphoma, small-cell melanoma, metastatic small-cell lung cancer (SCLC) to the skin and other small round-cell neoplasms involving the skin^3^. MCC can also be misdiagnosed as basal-cell carcinoma^128^. Thus, immunohistochemical investigations are necessary for diagnostic confirmation. MCCs typically express neuroendocrine markers, including chromogranin A and/or synaptophysin, although these are not specific markers. Cytokeratin 20 (CK20) is expressed focally or diffusely in most MCCs, typically in a paranuclear dot-like pattern, a cytoplasmic and/or membranous pattern, or both. Staining for neurofilament, another intermediate filament, can also have a paranuclear dot-like pattern. CK20 expression and the paranuclear dot-like pattern of intermediate filament staining are highly suggestive of MCC^3^. Synoptic reporting of newly diagnosed lesions facilitates treatment decisions and prognostic studies and as a minimum should include gross tumour size (in centimetres), peripheral and deep margin status and the extent of both lymphovascular invasion and extracutaneous extension (bone, muscle, fascia and/or cartilage involvement)^129^.

MCC is morphologically indistinguishable from metastatic SCLC. Immunohistochemical markers that enable distinction between these two entities include CK20, TTF1, MCPyV LT and neurofilament proteins. The MCC immunophenotype is CK20^+^, LT^+/−^, neurofilament^+^ and TTF1^−^. Metastatic SCLC to the skin is CK20^−^, LT^−^, neurofilament^−^ and TTF1^+^ (ref.^3^). However, expression patterns vary, and no single marker used in isolation is sufficiently sensitive or specific for the robust distinction of MCC from metastatic SCLC. Thus, a panel of markers is necessary, particularly for the diagnosis of challenging cases such as CK20-negative MCC. In addition, certain non-lung small-cell carcinomas (SmCCs), such as parotid and uterine cervical primary tumours, also frequently express CK20 and might, therefore, be more challenging to distinguish from MCC, especially in the setting of metastatic MCC from an unknown primary site^3^. Distinguishing between primary parotid SmCC and metastatic MCC of unknown primary is especially problematic, as the parotid is frequently a site of regional MCC metastasis, and primary parotid SmCC is rare^130^. As NGS-based analyses enter more widespread use, MCPyV detection combined with analysis of mutational signatures might be useful for distinguishing MCC (MCPyV-positive or with ultraviolet signature mutations) from metastatic SCLC (MCPyV-negative with smoking signature mutations)^83^. For example, ultraviolet signature mutations detected in a parotid gland tumour confirmed a diagnosis of metastatic MCC of unknown primary origin rather than a primary parotid neuroendocrine carcinoma^5^.

Patients with MCC have the potential to develop distant cutaneous metastases. In patients who present with a rare second cutaneous MCC that is spatially and temporally separated from the initial primary MCC, such that the tumour is clinically designated a second primary, investigations of clonality based on copy number alterations, mutations and/or MCPyV sequencing might be useful in distinguishing metastatic disease from a second primary MCC^79,131^.

### Prognostic findings

The most clinically relevant prognostic parameters for MCC include tumour size (defined as maximum tumour diameter) and the presence of locoregional or distant metastases. These factors form the basis of the American Joint Committee on Cancer staging system for MCC^132,133^. Larger primary tumour size correlates with an increased risk of metastatic disease, although MCCs of any size confer a substantial risk of occult metastasis, thus supporting the use of sentinel lymph node biopsy sampling for all patients with clinically node-negative disease^134^. In a large-cohort study with results published in 2015, Breslow depth (the distance in millimetres between the top of the granular layer of the epidermis and the deepest point of tumour invasion) was found to be an independent prognostic risk factor for overall survival and sentinel lymph node positivity and was moderately correlated with tumour diameter (*r* = 0.53, *P* < 0.0001)^135^. Additional features of the primary tumour, such as lymphovascular invasion and tumour growth pattern, have also been proposed to have prognostic significance but have not been evaluated on a large scale^136–138^. The presence of clinically detectable nodal disease is associated with worse outcomes than the presence of microscopic metastases^132^. Other findings associated with a worse prognosis include a sheet-like pattern of involvement in lymph node metastases and an increasing number of lymph nodes with metastatic involvement^133,139^.

Owing to the difficulties in predicting aggressive disease on the basis of clinical and morphological findings alone, prognostic biomarkers for MCC have been an area of intense investigation. Studies of the prognostic role of MCPyV status have, thus far, had mixed results, predominantly finding either a worsened prognosis in patients with VN-MCC tumours or no difference relative to VP-MCC tumours^140^. The largest study to date to incorporate both immunohistochemical and PCR-based evaluations of MCPyV status found a better prognosis in patients with VP-MCCs^73^. Nevertheless, both VP-MCCs and VN-MCCs can have clinically aggressive and fatal courses.

For patients with VP-MCC, MCPyV serology can be informative for prognosis and can, therefore, guide disease management. Higher anti-VP1 antibody titres and the presence of anti-ST antibodies in the serum at diagnosis have been associated with more favourable outcomes^141,142^. A finding of persistent or re-emergent anti-ST antibodies in sera has been associated with a poor prognosis and a high risk of disease recurrence^141^.

Interestingly, patients presenting with nodal or presumed metastatic MCC with no identifiable skin lesions can have better outcomes than patients with disease of the same stage with a known primary tumour^119,127,132^. An antitumour immune response has been proposed to underlie both the primary tumour regression and improved patient outcomes in such patients. Analogously, data from multiple studies have confirmed the importance of immune competence as a determinant of prognosis in patients with MCC; immunosuppressed patients (including those with chronic lymphocytic leukaemia) have not only a higher incidence of MCC but also markedly worse survival outcomes^125,143^.

Biomarkers associated with immune competence have been extensively investigated in patients with MCC. Patterns of immune-cell infiltration might reflect both MCPyV status and disease outcomes. Relative to VN-MCCs, VP-MCCs are more likely to be associated with evidence of a brisk inflammatory response and an increased number of CD8^+^ T cells^144–146^. Immunological findings that have been correlated with an improved prognosis among patients with MCC include increased CD8^+^ T cells (either tumour-infiltrating T cells or those located at the tumour periphery), tumour-infiltrating MCPyV-specific T cells and tumour programmed cell death 1 ligand 1 (PD-L1) expression^111,145–149^.

Additional markers proposed to have prognostic significance in MCC include tumour protein 63 (p63), EZH2, survivin (nuclear pattern), CD34 (vascular density), VEGF, vascular E-selectin, sonic hedgehog protein, phosphorylated signal transducer and activator of transcription 5B, cell adhesion molecule 1 and myelin and lymphocyte protein, among others^3^. *TP53* mutations have also been shown to have prognostic implications^6^, although whether this effect is independent of MCPyV status remains unknown.

### Management of MCC

Surgical excision with 1–2 cm margins, typically followed by radiotherapy, are the mainstays of the management of primary MCCs. Adjuvant radiotherapy to the primary tumour site is often recommended; however, the morbidity risks associated with radiotherapy can be avoided with a low local recurrence rate in a subset of patients (such as those with tumours <2 cm in size without other adverse prognostic features)^150^.

Owing to the risk of occult nodal disease, sentinel lymph node biopsy sampling is recommended by the National Comprehensive Cancer Network (NCCN) for patients without clinically detectable metastatic disease^8^. Any size of metastatic deposit is currently considered positive with regard to nodal staging; therefore, immunohistochemistry for broad-spectrum cytokeratins and/or CK20 is routinely used to improve the detection of micrometastases in sentinel lymph nodes^3,151^. The NCCN recommends the management of clinically detectable or occult nodal disease using imaging investigations for distant metastases, followed by lymph node dissection and/or radiotherapy to the nodal basin^129^.

Systemic therapies for patients with MCC have traditionally included chemotherapy, such as platinum-based drugs, taxanes, anthracyclines and etoposide. Owing to a lack of durable responses and no established effects on survival, chemotherapy is currently considered to have a palliative role^152^.

In the past 2 years, the anti-PD-L1 antibody avelumab has been approved as a therapy for patients with metastatic MCC by the FDA, the European Medicines Agency (EMA), Swissmedic and the Japanese Ministry of Health, Labor and Welfare^153^. Other immunotherapies for MCC have also demonstrated efficacy in clinical trials^154^. The success of immune checkpoint inhibition (ICI) is a milestone in the management of advanced-stage MCC. However, not all patients have durable responses to ICI. Furthermore, patients requiring immunosuppression as recipients of solid organ transplants or those with autoimmune disease might not be optimal candidates for ICI. Therefore, predicting and improving responsiveness to immunotherapy and identifying alternative therapies for patients in whom ICI is contraindicated and/or ineffective are both current research priorities in patients with MCC.

## Investigational therapies in MCC

### Immunotherapies

Several observations suggested that ICI would be effective in patients with MCC. Spontaneous regression of MCC is an uncommon but well-documented event, presumably owing to immune activation. Patients with T cell-infiltrated MCCs have better outcomes than those with tumours lacking infiltrating lymphocytes. The abscopal effect of irradiation (in which localized radiotherapy is associated with regression of additional tumour foci located outside of the treatment field) has also been observed in patients with MCC, suggesting a modulated immune response^155^. MCCs have a higher incidence and worse prognosis in the setting of immune compromise. Finally, MCCs express either MCPyV antigens or ultraviolet-mutation-associated neoantigens, providing putative targets for antitumour immunity^9^ (Fig. 3).

**Fig. 4:**
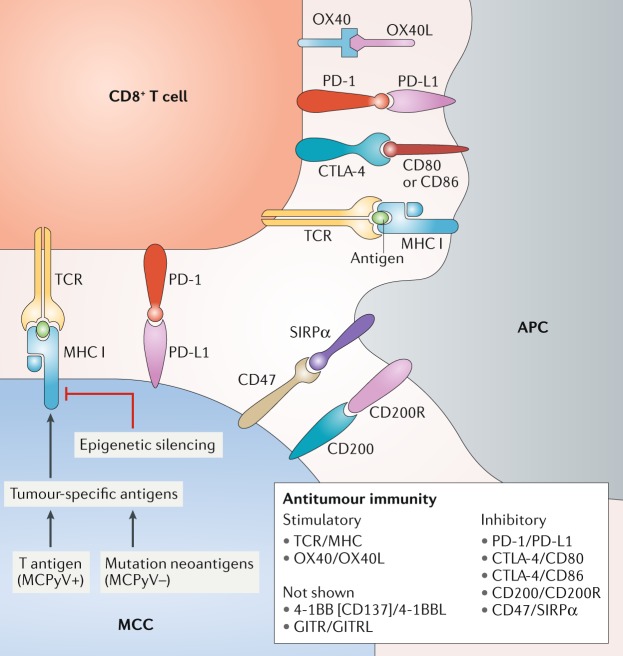
Signalling pathways that modulate antitumour immunity. Antitumour immunity is modulated by signalling molecules expressed on immune cells, including T cells and antigen-presenting cells (APCs), as well as Merkel cell carcinoma (MCC) cells. Adaptive antitumour immunity is primarily mediated by the presentation of tumour antigens on MHCs of either tumour cells or APCs. In the context of MCC, tumour-associated antigens can be either viral protein products in Merkel cell polyomavirus (MCPyV)-positive MCC or, in MCPyV-negative tumours, neoantigens resulting from somatic mutations. In virus-positive-MCC tumour cells, antigen presentation by MHC complexes is commonly suppressed through epigenetic silencing. Multiple signalling pathways have the potential to either stimulate (such as OX40–OX40L) or suppress antitumour immunity (such as programmed cell death protein 1 (PD-1)– programmed cell death protein 1 ligand 1 (PD-L1)). These various immune signalling pathways should be considered potential therapeutic targets. CTLA-4, cytotoxic T antigen 4; GITR, glucocorticoid-induced TNFR-related protein; SIRP α, signal-regulatory protein α; TCR, T cell receptor.

Both the innate and the adaptive arms of the immune system can have antitumour effects. Adaptive antitumour immunity is mediated by the activation of effector T cells by antigen-presenting cells and is modulated by proteins expressed on the surface of both immune cells and tumour cells (Fig. 4). Activation of the immune system can be stimulated by multiple signalling pathways, including the OX40–OX40L and CD137–4-1BBL axes. Other signalling pathways (including the cytotoxic T lymphocyte antigen 4 (CTLA-4) and programmed cell death protein 1 (PD-1)–PD-L1 immune checkpoints) suppress immune responses and can be exploited by tumour cells as a mechanism of immune evasion^156^. Thus, immunotherapies promote antitumour immunity either by activating stimulatory pathways or by suppressing inhibitory pathways^156^. Of these, inhibition of the PD-1–PD-L1 immune checkpoint has been intensely investigated in MCC. Several open-label phase II clinical trials involving anti-PD-1 or anti-PD-L1 antibodies have demonstrated durable responses to these agents in a subset of patients with MCC. In 88 patients with metastatic MCC who had previously received chemotherapy, treatment with the anti-PD-L1 antibody avelumab resulted in a 32.8% objective response rate (ORR), including complete — and durable — responses in 9.1% of patients^157^ and an associated improvement in quality of life^158^. In a study involving 26 patients, treatment with the anti-PD-1 antibody pembrolizumab resulted in a 56% ORR, including complete responses in 15.4% of patients, as first-line systemic therapy^154^. The anti-PD-1 antibody nivolumab has also shown promise^9,159,160^. In all of these studies, patients responded regardless of the viral status of their tumours, although the cohort sizes were too small to enable rigorous comparisons between VP-MCCs and VN-MCCs. Avelumab is currently the only FDA-approved treatment of metastatic MCC^153^. Investigations of the efficacy of both avelumab and nivolumab as adjuvant therapies for patients without detectable tumour material after surgery are currently ongoing (NCT03271372 and NCT02196961).

Several immunotherapies that act through mechanisms other than inhibition of PD-1 or PD-L1 are currently under investigation for MCC (Supplementary Table 1). Therapeutic combinations including anti-CTLA-4 antibodies are an area of active investigation^8^. In a phase I trial (NCT01307267), utomilumab, which might stimulate antitumour immunity by acting as an agonist of CD137 (4-1BB), has been shown to be well tolerated, with preliminary evidence of antitumour activity^161^. Proof of principle that adoptive cell transfer using in vitro expanded autologous antitumour T cells in combination with IFNβ and radiotherapy (NCT01758458) was demonstrated by a finding of tumour regression in one patient with metastatic MCC^162^. A phase I/II trial combining adoptive cell transfer with IL-2 and avelumab after conditioning the tumour with radiotherapy or IFNβ is currently underway (NCT02584829)^8,163^. DNA vaccines are effective in mice implanted with B16 murine melanoma cells expressing LT or ST^164,165^. The cutaneous location of primary and satellite and/or in transit MCC renders these tumours amenable to injections of local therapies. The efficacy of intratumoural administration of IL-12 cDNA delivered via electroporation was investigated in a phase II trial involving 12 patients with MCC, and disease regression was observed in 12 of 30 treated lesions^166^. Finally, several phase II trials (NCT02819843 and NCT02978625) are currently recruiting patients to determine the safety and efficacy of intratumoural injections of the modified oncolytic herpesvirus talimogene laherparepvec^163^.

Combinations of immunotherapies with traditional treatments might also be effective in patients with MCC. Downregulation of MHC class I (MHC I) expression in MCC can be reversed by radiotherapy, IFNβ and chemotherapies, potentially increasing the sensitivity of tumours to subsequent immunotherapy^112,162^. The histone deacetylase inhibitor vorinostat in combination with the chemotherapeutic agent mithramycin A can also upregulate MHC I expression in MCC cell lines and xenografts^113^. Radiotherapy also has the potential to induce the release of tumour-associated antigens and increase inflammation, thus further synergizing with immunotherapy^8^.

### Targeted therapies

Alternatives to ICI are needed for patients with advanced-stage MCC who are immunosuppressed (and thus ineligible) or who do not respond to ICI. Several types of targeted therapies have been investigated in MCC cell lines and xenograft models, and some are undergoing further testing in early phase clinical trials.

YM155, a small-molecule inhibitor of the antiapoptotic protein survivin, is a potent inducer of cell death in VP-MCC cell lines and xenografts^49,167^. Similarly, a small-molecule inhibitor of the apoptosis regulator BCL-2 family proteins, ABT-263, has been shown to induce apoptosis in the majority of MCC cell lines tested, regardless of MCPyV status^168^. Moreover, BCL-2 antisense oligonucleotides were effective in xenograft models^169^ but failed to show benefit in a phase II trial involving 12 patients with MCC^170^.

MCCs express somatostatin receptors; therefore, somatostatin analogues are being investigated for both molecular imaging^171,172^ and treatment of patients with MCC (NCT01204476, NCT01652547, NCT02351128, NCT02936323 and NCT03167164)^8^. Hedgehog signalling has a role in the development of nonmalignant Merkel cells^173–175^, although MCC cell lines are not sensitive to Hedgehog inhibitors^99^.

In line with the observation of recurrent activation of the PI3K pathway in MCC tumours, preclinical and limited clinical data support the efficacy of PI3K–mTOR pathway inhibition in MCC: MCC cell lines are sensitive to inhibition of PI3K and mTOR^84,85,176,177^, and a complete response to the PI3Kδ inhibitor idelalisib has been reported in one patient, although the response might have been modified by concurrent use of radiotherapy^178^. Several clinical trials are investigating the safety and efficacy of mTOR inhibition in patients with MCC (NCT01155258, NCT02514824, NCT00655655 and NCT01204476)^163^ (Supplementary Table 1). In VP-MCCs with wild-type *TP53*, investigations of tumour biology suggest that MDM2 (HDM2) inhibitors can be effective^179^.

In general, activating tyrosine kinase mutations are not detectable in MCCs and, thus far, there has been little evidence that tyrosine kinase inhibition is an effective treatment approach for patients with MCC. Clinical remission following treatment with imatinib has been reported in one patient^180^, although a phase II trial failed to show any consistent benefit with this approach^181^. Pazopanib inhibits several tyrosine kinases, including VEGFR, PDGFR, FGFR and KIT; this agent has been associated with clinical benefit in case reports^182^ and was investigated in a trial in the UK^8^. A clinical trial involving the multikinase inhibitor cabozantinib in patients with advanced-stage, platinum-refractory MCC was terminated prematurely owing to poor tolerability and a lack of responses in the first eight patients treated^183^. Of note, evidence for ERK activation in MCCs is lacking^88^, and *HRAS* mutations do not render MCC cell lines responsive to MEK inhibition^7^.

## Consensus recommendations

On 5–6 March 2018, over 50 participants (Supplementary Box 1) gathered at the US National Cancer Institute, NIH, for the International Workshop on Merkel Cell Carcinoma Research (IWMCC). Scientists from academic, government and industry backgrounds, along with regulatory and patient representatives, came together to discuss the state of MCC research, identify high-priority research directions and develop research strategies. The workshop addressed basic, translational and clinical research with the goal of improving the outcomes of patients with MCC. Consensus statements and top research priorities identified by the IWMCC Working Group are presented below. An extended list of research questions considered at the Workshop can be found in Supplementary Box 2.

## Basic science

Key basic research questions for MCC relate to the MCC cell of origin, distinctions between VP-MCC and VN-MCC, and MCPyV biology (Box 1). The MCC cell of origin remains unknown and might be of a very different lineage to that suggested by the tumour phenotype. Furthermore, VP-MCC and VN-MCC might have distinct cells of origin.

Fundamental questions remain regarding the biology of MCPyV in human infection and tumorigenesis. The cellular reservoirs and mechanisms of transmission of infectious, wild-type MCPyV remain unknown. T antigens and other viral gene products have been shown to have diverse functions involving numerous cellular partners. In particular, the numerous activities and effects of ST are incompletely elucidated. The identity of the specific viral protein or proteins that are critical for viral latency, replication, transformation and maintaining an oncogenic phenotype is not well understood. Identification of the essential tumour-promoting pathways has implications for effective therapeutic targeting of VP-MCCs and might provide an explanation for the apparently unique status of MCPyV as the only polyomavirus with established oncogenic effects in humans.

Despite molecular differences, VP-MCCs and VN-MCCs are almost indistinguishable, both clinically and in their response to ICI. Common putative driver mutations (in *RB1* and *TP53*) are present in VN-MCCs. Studies comparing the biology of VN-MCCs to VP-MCCs might ultimately determine a minimum set of signalling pathways that must be disrupted — either by mutation or by viral infection — to initiate MCC tumorigenesis. Parallels can be drawn with head and neck squamous cell carcinoma, which also has a mixed viral and non-viral aetiology. Studies involving mouse models of MCC might help to address several key questions including the cell of origin, which viral T antigens (and T antigen functional domains) are sufficient to generate VP-MCC and which genomic changes are sufficient to generate VN-MCC.

Box 1 High-priority research questions for MCC **Key basic science questions**

- What is the cell of origin of Merkel cell carcinoma (MCC)?
- How does the MCC tumour microenvironment contribute to pathogenesis and immune evasion?
- What are the relative contributions of small T antigen (ST) and large T antigen (LT) to tumour initiation and maintenance?
- How do we most accurately model MCC (virus-positive MCC (VP-MCC) and virus-negative MCC (VN-MCC)) in mice, including features such as immune response and evasion, invasion and metastasis?
- What cell types harbour productive Merkel cell polyomavirus (MCPyV) infection?

**Key translational research questions**

- What are the potential therapeutic targets in VN-MCC and VP-MCC?
- Do genomic alterations — either global (such as mutation burden) or specific (such as *PIK3CA* mutations) — have implications for prognosis and/or therapeutic response?
- What is the optimal MCPyV detection method?
- What is the clinical value of distinguishing between VP-MCC and VN-MCC?

**Key clinical questions in MCC**

- What alternatives to immune checkpoint inhibition (ICI) are effective in nonresponders and/or patients who are ineligible for such treatments?
- What markers and/or tumour characteristics predict responsiveness to ICI?
- What treatment combinations will enhance the efficacy of ICI for MCC?
- What is the optimal role of radiotherapy in patients with MCC?
- How can clinical trials be optimized to maximize collaboration and minimize competition for eligible patients with this rare tumour?

## Translational science

Great progress has been made with ICI such that approximately half of all patients with advanced-stage MCC benefit from anti-PD-1 or anti-PD-L1 antibodies. However, many patients have either primary or acquired resistance to these agents. Combination immunotherapy approaches must be explored, although patients with MCC might also benefit from targeted approaches that synergize with immunotherapies or could be used in patients who do not respond to ICI. Unbiased small-molecule screens for agents that selectively affect VP-MCC or VN-MCC cell lines are currently ongoing. The wild-type status of *TP53* in most VP-MCC tumours suggests that small molecules that result in the activation of TP53, such as MDM2 antagonism with a nutlin-like agent^184^, can be effective and could also synergize with other therapies. Therapies targeting survivin or BCL-2 might also be effective, owing to the biology of most MCCs. Studies of these agents and others could be facilitated by the use of patient-derived xenograft models. Patient-derived xenografts are generally more easily established from MCC tumour specimens than are new cell lines. Many patients with VP-MCCs do not respond to ICI and, therefore, the probable defects in the number, diversity and avidity of MCPyV-specific T cells merit further investigation. Future research should consider therapeutic vaccines as neoadjuvant therapies for patients with MCC, such as using vaccinia viruses to deliver inactivated viral oncoproteins, as has been demonstrated in mouse models of HPV-driven pre-malignant disease^185^. Despite the striking molecular differences between VP-MCC and VN-MCC, a role of MCPyV status in guiding the clinical management of MCC has not yet been established. Translational investigations would benefit from routinely determining and reporting the MCPyV status of all tumours.

## Clinical science

The consensus on the clinical knowledge gaps and research priorities in MCC clusters around three major themes: therapeutics, biomarkers and infrastructure. The current armamentarium for the management of MCC includes surgery, radiation therapy, cytotoxic chemotherapy, ICI with anti-PD-1 or anti-PD-L1 antibodies and experimental therapies administered in clinical trials. The advent of anti-PD-1 and anti-PD-L1 antibodies has provided great benefit for some patients with MCC; however, it remains too early to determine the long-term outcomes of these patients. Moreover, subsets of patients are either refractory to anti-PD-1 or anti-PD-L1 antibodies or develop acquired resistance over time. Immunosuppression, previous solid organ and/or haematological stem cell transplantation and haematological malignancies are known risk factors for MCC. Patients with these risk factors have been routinely excluded from clinical trials and thus the optimal therapeutic strategy for these individuals is unclear. Finally, conventional cytotoxic chemotherapy and radiation therapy are active therapies that predate the introduction of anti-PD-1 and anti-PD-L1 antibodies, and the best use of these agents in conjunction with immunotherapy remains to be determined.

The fact that MCC is either polyomavirus-positive (VP-MCC) or negative (VN-MCC) presents both unique opportunities and a therapeutic conundrum. At this time, MCC viral status does not help stratify patients into those who are more or less likely to respond to any specific therapy, given the present state of our knowledge and the available therapeutic options. We anticipate that targetable oncogenic drivers, at least in those with VP-MCC, are within reach. VN-MCC tumours generally have a molecular signature similar to that of the prototypical pattern seen in melanoma, characterized by ultraviolet-induced DNA damage and the presence of tumour-associated neoantigens. Thus, clinical developments might likewise follow the path taken by other immunotherapy-responsive tumours, including melanoma.

The clinical management of MCC would be improved by the creation of a collaborative infrastructure that enables formal information sharing and the rapid design and implementation of clinical trials. This aspect is particularly critical in a disease with such a low prevalence. Current understanding of the natural history of MCC and outcomes related to therapeutic interventions is scant, fractionated among individual investigators, and thus a common standard is lacking. To further our knowledge of the natural history of MCC, the implications of the various prognostic factors, predictors of drug response, implications of MCPyV status in therapeutic response and rapid clinical trial design and implementation, a collaborative multi-institutional effort is needed.

## Conclusions

Substantial advances have been made in our understanding of MCC biology, diagnosis and therapy; however, major research challenges remain. A striking molecular dichotomy exists between VP-MCC and VN-MCC, although the biological and clinical consequences of this difference — including critical pathways for tumorigenesis — are incompletely understood. Furthermore, the MCC cell of origin remains unknown. Finally, continued progress in improving patient outcomes will require the validation of additional therapeutic options in order to augment the gains provided by surgery, radiotherapy and immunotherapy.

## Supplementary information

Supplementary Information

## References

[1] Toker C (1972). Trabecular carcinoma of the skin. Arch. Dermatol..

[2] Agelli M, Clegg LX, Becker JC, Rollison DE (2010). The etiology and epidemiology of merkel cell carcinoma. Curr. Probl. Cancer.

[3] Harms PW (2017). Update on Merkel cell carcinoma. Clin. Lab. Med..

[4] Feng H, Shuda M, Chang Y, Moore PS (2008). Clonal integration of a polyomavirus in human Merkel cell carcinoma. Science.

[5] Harms PW (2015). The distinctive mutational spectra of polyomavirus-negative Merkel cell carcinoma. Cancer Res..

[6] Goh G (2016). Mutational landscape of MCPyV-positive and MCPyV-negative Merkel cell carcinomas with implications for immunotherapy. Oncotarget.

[7] Wong SQ (2015). UV-associated mutations underlie the etiology of MCV-negative Merkel cell carcinomas. Cancer Res..

[8] Cassler NM, Merrill D, Bichakjian CK, Brownell I (2016). Merkel cell carcinoma therapeutic update. Curr. Treat. Opt. Oncol..

[9] Becker JC (2017). Merkel cell carcinoma. Nat. Rev. Dis. Primers.

[10] Tolstov YL (2009). Human Merkel cell polyomavirus infection II. MCV is a common human infection that can be detected by conformational capsid epitope immunoassays. Int. J. Cancer.

[11] Tolstov YL (2011). Asymptomatic primary Merkel cell polyomavirus infection among adults. Emerg. Infect. Dis..

[12] Viscidi RP (2011). Age-specific seroprevalence of Merkel cell polyomavirus, BK virus, and JC virus. Clin. Vaccine Immunol..

[13] Nicol JT (2013). Age-specific seroprevalences of Merkel cell polyomavirus, human polyomaviruses 6, 7 , and 9, and trichodysplasia spinulosa-associated polyomavirus. Clin. Vaccine Immunol..

[14] Martel-Jantin C (2013). Merkel cell polyomavirus infection occurs during early childhood and is transmitted between siblings. J. Clin. Virol..

[15] Hashida Y (2016). Ecology of Merkel cell polyomavirus in healthy skin among individuals in an Asian cohort. J. Infect. Dis..

[16] Pastrana DV (2009). Quantitation of human seroresponsiveness to Merkel cell polyomavirus. PLOS Pathog..

[17] Carter JJ (2009). Association of Merkel cell polyomavirus-specific antibodies with Merkel cell carcinoma. J. Natl Cancer Inst..

[18] Touze A (2010). Generation of Merkel cell polyomavirus (MCV)-like particles and their application to detection of MCV antibodies. J. Clin. Microbiol..

[19] Goh S, Lindau C, Tiveljung-Lindell A, Allander T (2009). Merkel cell polyomavirus in respiratory tract secretions. Emerg. Infect. Dis..

[20] Kantola K (2009). Merkel cell polyomavirus DNA in tumor-free tonsillar tissues and upper respiratory tract samples: implications for respiratory transmission and latency. J. Clin. Virol..

[21] Pancaldi C (2011). Merkel cell polyomavirus DNA sequences in the buffy coats of healthy blood donors. Blood.

[22] Moustafa A (2017). The blood DNA virome in 8,000 humans. PLOS Pathog..

[23] Fukumoto H, Sato Y, Hasegawa H, Katano H (2013). Frequent detection of Merkel cell polyomavirus DNA in sera of HIV-1-positive patients. Virol. J..

[24] Mertz KD, Junt T, Schmid M, Pfaltz M, Kempf W (2010). Inflammatory monocytes are a reservoir for Merkel cell polyomavirus. J. Invest. Dermatol..

[25] Matsushita M (2013). Detection of Merkel cell polyomavirus in the human tissues from 41 Japanese autopsy cases using polymerase chain reaction. Intervirology.

[26] Mancuso G (2017). Frequent detection of Merkel cell polyomavirus DNA in tissues from 10 consecutive autopsies. J. Gen. Virol..

[27] Schowalter RM, Pastrana DV, Pumphrey KA, Moyer AL, Buck CB (2010). Merkel cell polyomavirus and two previously unknown polyomaviruses are chronically shed from human skin. Cell Host Microbe.

[28] Liu W (2016). Identifying the target cells and mechanisms of Merkel Cell polyomavirus infection. Cell Host Microbe.

[29] Schowalter RM, Reinhold WC, Buck CB (2012). Entry tropism of BK and Merkel cell polyomaviruses in cell culture. PLOS ONE.

[30] Kwun HJ, Chang Y, Moore PS (2017). Protein-mediated viral latency is a novel mechanism for Merkel cell polyomavirus persistence. Proc. Natl Acad. Sci. USA.

[31] DeCaprio JA (2017). Merkel cell polyomavirus and Merkel cell carcinoma. Philos. Trans. R . Soc. B. Biol. Sci..

[32] Carter JJ (2013). Identification of an overprinting gene in Merkel cell polyomavirus provides evolutionary insight into the birth of viral genes. Proc. Natl Acad. Sci. USA.

[33] Lee S (2011). Identification and validation of a novel mature microRNA encoded by the Merkel cell polyomavirus in human Merkel cell carcinomas. J. Clin. Virol..

[34] Theiss JM (2015). A comprehensive analysis of replicating Merkel cell polyomavirus genomes delineates the viral transcription program and suggests a role for mcv-miR-M1 in episomal persistence. PLOS Pathog..

[35] Schowalter RM, Buck CB (2013). The Merkel cell polyomavirus minor capsid protein. PLOS Pathog..

[36] Seo GJ, Chen CJ, Sullivan CS (2009). Merkel cell polyomavirus encodes a microRNA with the ability to autoregulate viral gene expression. Virology.

[37] Shuda M (2008). T antigen mutations are a human tumor-specific signature for Merkel cell polyomavirus. Proc. Natl Acad. Sci. USA.

[38] Starrett GJ (2017). Merkel cell polyomavirus exhibits dominant control of the tumor genome and transcriptome in virus-associated Merkel cell carcinoma. mBio.

[39] Duncavage EJ (2011). Hybrid capture and next-generation sequencing identify viral integration sites from formalin-fixed, paraffin-embedded tissue. J. Mol. Diagn..

[40] Laude HC (2010). Distinct merkel cell polyomavirus molecular features in tumour and non tumour specimens from patients with merkel cell carcinoma. PLOS Pathog..

[41] Martel-Jantin C (2012). Genetic variability and integration of Merkel cell polyomavirus in Merkel cell carcinoma. Virology.

[42] Sastre-Garau X (2009). Merkel cell carcinoma of the skin: pathological and molecular evidence for a causative role of MCV in oncogenesis. J. Pathol..

[43] Kwun HJ (2009). The minimum replication origin of merkel cell polyomavirus has a unique large T-antigen loading architecture and requires small T-antigen expression for optimal replication. J. Virol..

[44] Li J (2013). Merkel cell polyomavirus large T antigen disrupts host genomic integrity and inhibits cellular proliferation. J. Virol..

[45] Houben R (2010). Merkel cell polyomavirus-infected Merkel cell carcinoma cells require expression of viral T antigens. J. Virol..

[46] Houben R (2012). An intact retinoblastoma protein-binding site in Merkel cell polyomavirus large T antigen is required for promoting growth of Merkel cell carcinoma cells. Int. J. Cancer.

[47] Shuda M, Chang Y, Moore PS (2014). Merkel cell polyomavirus-positive Merkel cell carcinoma requires viral small T-antigen for cell proliferation. J. Invest. Dermatol..

[48] Houben R (2015). Characterization of functional domains in the Merkel cell polyoma virus Large T antigen. Int. J. Cancer.

[49] Arora R (2012). Survivin is a therapeutic target in Merkel cell carcinoma. Sci. Transl Med..

[50] Borchert S (2014). High-affinity Rb binding, p53 inhibition, subcellular localization, and transformation by wild-type or tumor-derived shortened Merkel cell polyomavirus large T antigens. J. Virol..

[51] Liu X (2011). Merkel cell polyomavirus large T antigen disrupts lysosome clustering by translocating human Vam6p from the cytoplasm to the nucleus. J. Biol. Chem..

[52] Wang X (2012). Bromodomain protein Brd4 plays a key role in Merkel cell polyomavirus DNA replication. PLOS Pathog..

[53] Shuda M, Kwun HJ, Feng H, Chang Y, Moore PS (2011). Human Merkel cell polyomavirus small T antigen is an oncoprotein targeting the 4E-BP1 translation regulator. J. Clin. Invest..

[54] Shuda M (2015). Merkel cell polyomavirus small T antigen induces cancer and embryonic Merkel cell proliferation in a transgenic mouse model. PLOS ONE.

[55] Verhaegen ME (2015). Merkel cell polyomavirus small T antigen is oncogenic in transgenic mice. J. Invest. Dermatol..

[56] Spurgeon ME, Cheng J, Bronson RT, Lambert PF, DeCaprio JA (2015). Tumorigenic activity of merkel cell polyomavirus T antigens expressed in the stratified epithelium of mice. Cancer Res..

[57] Verhaegen ME (2017). Merkel cell polyomavirus small T antigen initiates Merkel cell carcinoma-like tumor development in mice. Cancer Res..

[58] Kwun HJ (2015). Restricted protein phosphatase 2A targeting by Merkel cell polyomavirus small T antigen. J. Virol..

[59] Abdul-Sada H (2017). The PP4R1 sub-unit of protein phosphatase PP4 is essential for inhibition of NF-kappaB by merkel polyomavirus small tumour antigen. Oncotarget.

[60] Stakaityte G (2017). Merkel cell polyomavirus small T antigen drives cell motility via Rho-GTPase-induced filopodia formation. J. Virol..

[61] Knight LM (2015). Merkel cell polyomavirus small T antigen mediates microtubule destabilization to promote cell motility and migration. J. Virol..

[62] Griffiths DA (2013). Merkel cell polyomavirus small T antigen targets the NEMO adaptor protein to disrupt inflammatory signaling. J. Virol..

[63] Kwun HJ (2013). Merkel cell polyomavirus small T antigen controls viral replication and oncoprotein expression by targeting the cellular ubiquitin ligase SCFFbw7. Cell Host Microbe.

[64] Shuda M (2015). CDK1 substitutes for mTOR kinase to activate mitotic cap-dependent protein translation. Proc. Natl Acad. Sci. USA.

[65] Bjornsti MA, Houghton PJ (2004). Lost in translation: dysregulation of cap-dependent translation and cancer. Cancer Cell.

[66] Cheng J (2017). Merkel cell polyomavirus recruits MYCL to the EP400 complex to promote oncogenesis. PLOS Pathog..

[67] Berrios C (2016). Merkel cell polyomavirus small T antigen promotes pro-glycolytic metabolic perturbations required for transformation. PLOS Pathog..

[68] Matsushita M (2014). A new in situ hybridization and immunohistochemistry with a novel antibody to detect small T-antigen expressions of Merkel cell polyomavirus (MCPyV). Diagn. Pathol..

[69] Wang L (2017). Age and gender associations of virus positivity in Merkel cell carcinoma characterized using a novel RNA in situ hybridization assay. Clin. Cancer Res..

[70] Rodig SJ (2012). Improved detection suggests all Merkel cell carcinomas harbor Merkel polyomavirus. J. Clin. Invest..

[71] Shuda M (2009). Human Merkel cell polyomavirus infection I. MCV T antigen expression in Merkel cell carcinoma, lymphoid tissues and lymphoid tumors. Int. J. Cancer.

[72] Haugg AM (2011). Fluorescence in situ hybridization confirms the presence of Merkel cell polyomavirus in chronic lymphocytic leukemia cells. Blood.

[73] Moshiri AS (2016). Polyomavirus-negative Merkel cell carcinoma: a more aggressive subtype based on analysis of 282 cases using multimodal tumor virus detection. J. Invest. Dermatol..

[74] Leroux-Kozal V (2015). Merkel cell carcinoma: histopathologic and prognostic features according to the immunohistochemical expression of Merkel cell polyomavirus large T antigen correlated with viral load. Hum. Pathol..

[75] Sihto H (2009). Clinical factors associated with Merkel cell polyomavirus infection in Merkel cell carcinoma. J. Natl Cancer Inst..

[76] Katano H (2009). Detection of Merkel cell polyomavirus in Merkel cell carcinoma and Kaposi’s sarcoma. J. Med. Virol..

[77] Eid M, Nguyen J, Brownell I (2017). Seeking standards for the detection of Merkel cell polyomavirus and its clinical significance. J. Invest. Dermatol..

[78] Paulson KG (2009). Array-CGH reveals recurrent genomic changes in Merkel cell carcinoma including amplification of L-Myc. J. Invest. Dermatol..

[79] Harms KL (2017). Multiple primary Merkel cell carcinoma: molecular profiling to distinguish genetically distinct tumors from clonally related metastases. JAMA Dermatol..

[80] Larramendy ML, Koljonen V, Bohling T, Tukiainen E, Knuutila S (2004). Recurrent DNA copy number changes revealed by comparative genomic hybridization in primary Merkel cell carcinomas. Mod. Pathol..

[81] Sahi H (2014). RB1 gene in Merkel cell carcinoma: hypermethylation in all tumors and concurrent heterozygous deletions in the polyomavirus-negative subgroup. Acta Pathol. Microbiol. Immunol. Scand..

[82] Cimino PJ (2014). Retinoblastoma gene mutations detected by whole exome sequencing of Merkel cell carcinoma. Mod. Pathol..

[83] Harms PW (2016). Next generation sequencing of cytokeratin 20-negative Merkel cell carcinoma reveals ultraviolet-signature mutations and recurrent TP53 and RB1 inactivation. Mod. Pathol..

[84] Nardi V (2012). Activation of PI3K signaling in Merkel cell carcinoma. Clin. Cancer Res..

[85] Hafner C (2012). Activation of the PI3K/AKT pathway in Merkel cell carcinoma. PLOS ONE.

[86] Veija T, Sarhadi VK, Koljonen V, Bohling T, Knuutila S (2016). Hotspot mutations in polyomavirus positive and negative Merkel cell carcinomas. Cancer Genet..

[87] Iwasaki T (2014). Comparison of Akt/mTOR/4E-BP1 pathway signal activation and mutations of PIK3CA in Merkel cell polyomavirus-positive and Merkel cell polyomavirus-negative carcinomas. Hum. Pathol..

[88] Houben R (2006). Absence of classical MAP kinase pathway signalling in Merkel cell carcinoma. J. Invest. Dermatol..

[89] Krasagakis K (2011). KIT receptor activation by autocrine and paracrine stem cell factor stimulates growth of merkel cell carcinoma in vitro. J. Cell. Physiol..

[90] Houben R (2007). Activation of the MAP kinase pathway induces apoptosis in the Merkel cell carcinoma cell line UISO. J. Invest. Dermatol..

[91] Khan AQ (2018). RAS-mediated oncogenic signaling pathways in human malignancies. Semin. Cancer Biol..

[92] Kartha RV, Sundram UN (2008). Silent mutations in KIT and PDGFRA and coexpression of receptors with SCF and PDGFA in Merkel cell carcinoma: implications for tyrosine kinase-based tumorigenesis. Mod. Pathol..

[93] Swick BL, Ravdel L, Fitzpatrick JE, Robinson WA (2008). Platelet-derived growth factor receptor alpha mutational status and immunohistochemical expression in Merkel cell carcinoma: implications for treatment with imatinib mesylate. J. Cutan. Pathol..

[94] Swick BL, Srikantha R, Messingham KN (2013). Specific analysis of KIT and PDGFR-alpha expression and mutational status in Merkel cell carcinoma. J. Cutan. Pathol..

[95] Andea AA (2010). Merkel cell carcinoma: correlation of KIT expression with survival and evaluation of KIT gene mutational status. Hum. Pathol..

[96] Lill C (2013). Expression of beta-catenin and cyclin D1 in Merkel cell carcinomas of the head and neck. Wien. Klin. Wochenschr..

[97] Liu S, Daa T, Kashima K, Kondoh Y, Yokoyama S (2007). The Wnt-signaling pathway is not implicated in tumorigenesis of Merkel cell carcinoma. J. Cutan. Pathol..

[98] Weeraratna AT, Houben R, O’Connell MP, Becker JC (2010). Lack of Wnt5A expression in Merkel cell carcinoma. Arch. Dermatol..

[99] Carroll TM (2017). Hedgehog signaling inhibitors fail to reduce Merkel cell carcinoma viability. J. Invest. Dermatol..

[100] Brunner M (2010). Expression of hedgehog signaling molecules in Merkel cell carcinoma. Head Neck.

[101] Kuromi T (2017). Association of expression of the hedgehog signal with Merkel cell polyomavirus infection and prognosis of Merkel cell carcinoma. Hum. Pathol..

[102] Sunshine JC, Jahchan NS, Sage J, Choi J (2018). Are there multiple cells of origin of Merkel cell carcinoma?. Oncogene.

[103] Daily K (2015). Assessment of cancer cell line representativeness using microarrays for Merkel cell carcinoma. J. Invest. Dermatol..

[104] Renwick N (2013). Multicolor microRNA FISH effectively differentiates tumor types. J. Clin. Invest..

[105] Haag T, Richter AM, Schneider MB, Jimenez AP, Dammann RH (2016). The dual specificity phosphatase 2 gene is hypermethylated in human cancer and regulated by epigenetic mechanisms. BMC Cancer.

[106] Lassacher A, Heitzer E, Kerl H, Wolf P (2008). p14ARF hypermethylation is common but INK4a-ARF locus or p53 mutations are rare in Merkel cell carcinoma. J. Invest. Dermatol..

[107] Richter AM (2013). Aberrant promoter hypermethylation of RASSF family members in Merkel cell carcinoma. Cancers.

[108] Helmbold P (2009). Frequent occurrence of RASSF1A promoter hypermethylation and Merkel cell polyomavirus in Merkel cell carcinoma. Mol. Carcinog..

[109] Harms KL (2017). Increased expression of EZH2 in Merkel cell carcinoma is associated with disease progression and poorer prognosis. Hum. Pathol..

[110] Miner AG (2014). Cytokeratin 20-negative Merkel cell carcinoma is infrequently associated with the Merkel cell polyomavirus. Mod. Pathol..

[111] Paulson KG (2011). Transcriptome-wide studies of merkel cell carcinoma and validation of intratumoral CD8+ lymphocyte invasion as an independent predictor of survival. J. Clin. Oncol..

[112] Paulson KG (2014). Downregulation of MHC-I expression is prevalent but reversible in Merkel cell carcinoma. Cancer Immunol. Res..

[113] Ritter C (2017). Epigenetic priming restores the HL A class-I antigen processing machinery expression in Merkel cell carcinoma. Sci. Rep..

[114] Zur Hausen A, Rennspiess D, Winnepenninckx V, Speel EJ, Kurz AK (2013). Early B cell differentiation in Merkel cell carcinomas: clues to cellular ancestry. Cancer Res..

[115] Murakami I (2014). Immunoglobulin expressions are only associated with MCPyV-positive Merkel cell carcinomas but not with MCPyV-negative ones: comparison of prognosis. Am. J. Surg. Pathol..

[116] Becker JC, zur Hausen A (2014). Cells of origin in skin cancer. J. Invest. Dermatol..

[117] Sell S (2010). On the stem cell origin of cancer. Am. J. Pathol..

[118] Sanchez-Martin D (2017). Evidence for a mesothelial origin of body cavity effusion lymphomas. J. Natl Cancer Inst..

[119] Paulson KG (2018). Merkel cell carcinoma: current US incidence and projected increases based on changing demographics. J. Am. Acad. Dermatol..

[120] Stang A, Becker JC, Nghiem P, Ferlay J (2018). The association between geographic location and incidence of Merkel cell carcinoma in comparison to melanoma: an international assessment. Eur. J. Cancer.

[121] Schadendorf D (2017). Merkel cell carcinoma: epidemiology , prognosis, therapy and unmet medical needs. Eur. J. Cancer.

[122] Youlden DR, Soyer HP, Youl PH, Fritschi L, Baade PD (2014). Incidence and survival for Merkel cell carcinoma in Queensland, Australia, 1993–2010. JAMA Dermatol..

[123] Paik JY (2011). Immunohistochemistry for Merkel cell polyomavirus is highly specific but not sensitive for the diagnosis of Merkel cell carcinoma in the Australian population. Hum. Pathol..

[124] Garneski KM (2009). Merkel cell polyomavirus is more frequently present in North American than Australian Merkel cell carcinoma tumors. J. Invest. Dermatol..

[125] Ma JE, Brewer JD (2014). Merkel cell carcinoma in immunosuppressed patients. Cancers.

[126] Heath M (2008). Clinical characteristics of Merkel cell carcinoma at diagnosis in 195 patients: the AEIOU features. J. Am. Acad. Dermatol..

[127] Vandeven NA (2017). Merkel cell carcinoma patients presenting without a primary lesion have elevated markers of immunity , higher tumor mutation burden and improved survival. Clin. Cancer Res..

[128] Ball NJ, Tanhuanco-Kho G (2007). Merkel cell carcinoma frequently shows histologic features of basal cell carcinoma: a study of 30 cases. J. Cutan. Pathol..

[129] Bichakjian CK (2018). Merkel cell carcinoma, version 1.2018, NCCN clinical practice guidelines in oncology. J. Natl Compr. Canc. Netw..

[130] Chernock RD, Duncavage EJ (2018). Proceedings of the NASHNP Companion Meeting, March 18th, 2018, Vancouver, BC, Canada: salivary neuroendocrine carcinoma—an overview of a rare disease with an emphasis on determining tumor origin. Head Neck Pathol..

[131] Schrama D (2010). Distinction of 2 different primary Merkel cell carcinomas in 1 patient by Merkel cell polyomavirus genome analysis. Arch. Dermatol..

[132] Harms KL (2016). Analysis of prognostic factors from 9387 Merkel cell carcinoma cases forms the basis for the new 8th edn AJCC staging system. Ann. Surg. Oncol..

[133] Iyer JG (2014). Relationships among primary tumor size, number of involved nodes, and survival for 8044 cases of Merkel cell carcinoma. J. Am. Acad. Dermatol..

[134] Schwartz JL (2011). Features predicting sentinel lymph node positivity in Merkel cell carcinoma. J. Clin. Oncol..

[135] Smith FO (2015). Both tumor depth and diameter are predictive of sentinel lymph node status and survival in Merkel cell carcinoma. Cancer.

[136] Andea AA, Coit DG, Amin B, Busam KJ (2008). Merkel cell carcinoma: histologic features and prognosis. Cancer.

[137] Lemos BD (2010). Pathologic nodal evaluation improves prognostic accuracy in Merkel cell carcinoma: analysis of 5823 cases as the basis of the first consensus staging system. J. Am. Acad. Dermatol..

[138] Al-Rohil RN (2018). Intratumoral and peritumoral lymphovascular invasion detected by D2-40 immunohistochemistry correlates with metastasis in primary cutaneous Merkel cell carcinoma. Hum. Pathol..

[139] Ko JS (2016). Histological pattern of Merkel cell carcinoma sentinel lymph node metastasis improves stratification of stage III patients. Mod. Pathol..

[140] Coursaget P, Samimi M, Nicol JT, Gardair C, Touze A (2013). Human Merkel cell polyomavirus: virological background and clinical implications. Acta Pathol. Microbiol. Immunol. Scand..

[141] Paulson KG (2017). Viral oncoprotein antibodies as a marker for recurrence of Merkel cell carcinoma: a prospective validation study. Cancer.

[142] Samimi M (2016). Prognostic value of antibodies to Merkel cell polyomavirus T antigens and VP1 protein in patients with Merkel cell carcinoma. Br. J. Dermatol..

[143] Paulson KG (2013). Systemic immune suppression predicts diminished Merkel cell carcinoma-specific survival independent of stage. J. Invest. Dermatol..

[144] Harms PW (2013). Distinct gene expression profiles of viral- and nonviral-associated merkel cell carcinoma revealed by transcriptome analysis. J. Invest. Dermatol..

[145] Sihto H (2012). Tumor infiltrating immune cells and outcome of Merkel cell carcinoma: a population-based study. Clin. Cancer Res..

[146] Lipson EJ (2013). PD-L1 expression in the Merkel cell carcinoma microenvironment: association with inflammation, Merkel cell polyomavirus and overall survival. Cancer Immunol. Res..

[147] Feldmeyer L (2016). Density , distribution, and composition of immune infiltrates correlate with survival in Merkel cell carcinoma. Clin. Cancer Res..

[148] Miller NJ (2017). Tumor-infiltrating Merkel cell polyomavirus-specific T cells are diverse and associated with improved patient survival. Cancer Immunol. Res..

[149] Paulson KG (2014). CD8+ lymphocyte intratumoral infiltration as a stage-independent predictor of Merkel cell carcinoma survival: a population-based study. Am. J. Clin. Pathol..

[150] Frohm ML (2016). Recurrence and survival in patients with Merkel cell carcinoma undergoing surgery without adjuvant radiation therapy to the primary site. JAMA Dermatol..

[151] Su LD (2002). Immunostaining for cytokeratin 20 improves detection of micrometastatic Merkel cell carcinoma in sentinel lymph nodes. J. Am. Acad. Dermatol..

[152] Nghiem P (2017). Systematic literature review of efficacy , safety and tolerability outcomes of chemotherapy regimens in patients with metastatic Merkel cell carcinoma. Future Oncol..

[153] Baker M, Cordes L, Brownell I (2018). Avelumab: a new standard for treating metastatic Merkel cell carcinoma. Expert Rev. Anticancer Ther..

[154] Nghiem PT (2016). PD-1 blockade with pembrolizumab in advanced Merkel-cell carcinoma. N. Engl. J. Med..

[155] Cotter SE (2011). Abscopal effect in a patient with metastatic Merkel cell carcinoma following radiation therapy: potential role of induced antitumor immunity. Arch. Dermatol..

[156] Torphy RJ, Schulick RD, Zhu Y (2017). Newly emerging immune checkpoints: promises for future cancer therapy. Int. J. Mol. Sci..

[157] Kaufman HL (2016). Avelumab in patients with chemotherapy-refractory metastatic Merkel cell carcinoma: a multicentre, single-group, open-label, phase 2 trial. Lancet Oncol..

[158] Kaufman HL, Hunger M, Hennessy M, Schlichting M, Bharmal M (2018). Nonprogression with avelumab treatment associated with gains in quality of life in metastatic Merkel cell carcinoma. Future Oncol..

[159] Walocko FM, Scheier BY, Harms PW, Fecher L A, Lao CD (2016). Metastatic Merkel cell carcinoma response to nivolumab. J. Immunother. Cancer.

[160] Mantripragada K, Birnbaum A (2015). Response to anti-PD-1 therapy in metastatic Merkel cell carcinoma metastatic to the heart and pancreas. Cureus.

[161] Segal NH (2018). Phase I study of single-agent utomilumab (PF-05082566), a 4-1BB/CD137 agonist, in patients with advanced cancer. Clin. Cancer Res..

[162] Chapuis AG (2014). Regression of metastatic Merkel cell carcinoma following transfer of polyomavirus-specific T cells and therapies capable of re-inducing HL A class-I. Cancer Immunol. Res..

[163] US National Library of Medicine. *ClinicalTrials.gov* https://clinicaltrials.gov/ct2/results?cond=Merkel+Cell+Carcinoma (2018).

[164] Gomez B (2013). Creation of a Merkel cell polyomavirus small T antigen-expressing murine tumor model and a DNA vaccine targeting small T antigen. Cell Biosci..

[165] Zeng Q (2012). Development of a DNA vaccine targeting Merkel cell polyomavirus. Vaccine.

[166] Bhatia S (2015). Intratumoral delivery of interleukin-12 DNA via in vivo electroporation leads to regression of injected and non-injected tumors in Merkel cell carcinoma: final results of a phase 2 study. Eur. J. Cancer.

[167] Dresang LR (2013). Response of Merkel cell polyomavirus-positive merkel cell carcinoma xenografts to a survivin inhibitor. PLOS ONE.

[168] Verhaegen ME (2014). Merkel cell carcinoma dependence on Bcl-2 family members for survival. J. Invest. Dermatol..

[169] Schlagbauer-Wadl H (2000). Bcl-2 antisense oligonucleotides (G3139) inhibit Merkel cell carcinoma growth in SCID mice. J. Invest. Dermatol..

[170] Shah MH (2009). G3139 (Genasense) in patients with advanced merkel cell carcinoma. Am. J. Clin. Oncol..

[171] Buder K (2014). Somatostatin receptor expression in Merkel cell carcinoma as target for molecular imaging. BMC Cancer.

[172] Kwekkeboom DJ, Hoff AM, Lamberts SW, Oei HY, Krenning EP (1992). Somatostatin analogue scintigraphy — a simple and sensitive method for the in vivo visualization of Merkel cell tumors and their metastases. Arch. Dermatol..

[173] Perdigoto CN (2016). Polycomb-mediated repression and Sonic Hedgehog signaling interact to regulate Merkel cell specification during skin development. PLOS Genet..

[174] Xiao Y (2016). A cascade of Wnt, Eda, and Shh signaling is essential for touch dome Merkel cell development. PLOS Genet..

[175] Xiao Y (2015). Neural Hedgehog signaling maintains stem cell renewal in the sensory touch dome epithelium. Proc. Natl Acad. Sci. USA.

[176] Kannan A (2016). Dual mTOR inhibitor MLN0128 suppresses Merkel cell carcinoma (MCC) xenograft tumor growth. Oncotarget.

[177] Lin Z, Mei H, Fan J, Yin Z, Wu G (2015). Effect of the dual phosphatidylinositol 3-kinase/mammalian target of rapamycin inhibitor NVP-BEZ235 against human Merkel cell carcinoma MKL-1 cells. Oncol. Lett..

[178] Shiver MB, Mahmoud F, Gao L (2015). Response to idelalisib in a patient with stage IV Merkel-cell carcinoma. N. Engl. J. Med..

[179] Houben R (2013). Mechanisms of p53 restriction in Merkel cell carcinoma cells are independent of the Merkel cell polyoma virus T antigens. J. Invest. Dermatol..

[180] Loader DE (2013). Clinical remission of Merkel cell carcinoma after treatment with imatinib. J. Am. Acad. Dermatol..

[181] Samlowski WE (2010). A phase II trial of imatinib mesylate in merkel cell carcinoma (neuroendocrine carcinoma of the skin): A Southwest Oncology Group study (S0331). Am. J. Clin. Oncol..

[182] Davids MS (2009). Response to a novel multitargeted tyrosine kinase inhibitor pazopanib in metastatic Merkel cell carcinoma. J. Clin. Oncol..

[183] Rabinowits G (2018). Cabozantinib in patients with advanced Merkel cell carcinoma. Oncologist.

[184] Secchiero P, Bosco R, Celeghini C, Zauli G (2011). Recent advances in the therapeutic perspectives of nutlin-3. Curr. Pharm. Des..

[185] Sun YY (2016). Local HPV recombinant vaccinia boost following priming with an HPV DNA vaccine enhances local HPV-specific CD8^+^ T cell–mediated tumor control in the genital tract. Clin. Cancer Res..

