# Supplementary material for: The biology and treatment of Merkel cell carcinoma: current understanding and research priorities
Source: Nat Rev Clin Oncol. 2018 Oct 4;15(12):763–76. doi: 10.1038/s41571-018-0103-2 (PMC6319370; doi:10.1038/s41571-018-0103-2)
Supplement: Supplementary file 1 — Supplementary Information [file 41571_2018_103_MOESM1_ESM.pdf]

nature REVIEWS CLINICAL ONCOLOGY

SUPPLEMENTARY INFORMATION

In format as provided by the authors

The biology and treatment of Merkel cell carcinoma: current understanding and research priorities

Paul W. Harms, Kelly L. Harms, Patrick S. Moore, James A. DeCaprio, Paul Nghiem, Michael K. K. Wong and Isaac Brownell, on behalf of the International Workshop on Merkel Cell Carcinoma Research (IWMCC) Working Group

https://doi.org/10.1038/s41571-018-0103-2

Supplementary Table 1. Investigational agents in MCC.

Intervention	Proposed Mechanism	Clinical Trial Phase(s)	Clinical Trial Number(s)
**PD-1/PD-L1 checkpoint inhibitors**			
Nivolumab	Anti-PD-1 antibody	Multiple phases 1 and 2 (Recruiting)	NCT02196961, NCT03071406, NCT03071757
Pembrolizumab	Anti-PD-1 antibody	Phase 2 (Active)	NCT02267603, NCT03304639
Avelumab	Anti-PD-L1 antibody	Expanded access, multiple phases 1-3 recruiting	NCT00655655, NCT02155647, NCT02584829, NCT03167164, NCT03271372
ABBV-181	Anti-PD1 antibody	Phase 1 (Recruiting)	NCT03000257
Durvalumab	Anti-PD-L1 antibody	Phase 1/2 (Recruiting)	NCT02643303
CK-301	Anti-PD-L1 antibody	Phase 1 (Recruiting)	NCT03212404
**CTLA4 inhibitors**			
Ipilimumab	Anti-CTLA4 antibody	Phase 2 (Recruiting)	NCT02196961
Tremelimumab	Anti-CTLA4 antibody	Phase 1/2 (Recruiting)	NCT02643303
**Other immune-based therapies**			
INCAGN01876	Anti-GITR agonist antibody	Multiple phase 1/2 (Recruiting)	NCT03277352, NCT03126110
INCAGN01949	Anti-OX40 agonist antibody	Phase 1/2 (Recruiting)	NCT03241173
Utomilumab	4-1BB agonist antibody	Phase 1 (Active)	NCT01307267
TTI-621	SIRPa-Fc fusion protein	Phase 1 (Recruiting)	NCT02890368
Adoptive immunotherapy	MCPyV TAg-specific polyclonal autologous CD8-positive T cells	Phase 1/2 (Recruiting)	NCT02584829 (previously NCT01758458)
Interferon Beta		Phase 1/2 (Recruiting)	NCT02584829
Poly ICLC	Stimulation of cytotoxic cytokines	Phase 1/2 (Recruiting)	NCT02643303
ALT-803	IL-15 agonist	Phase 1/2 (Not yet recruiting)	NCT03167164
GI-6301	Brachyury-expressing yeast vaccine	Phase 1/2 (Not yet recruiting)	NCT03167164
NK cell transfer	NK cell transfer	Multiple (phase 1/2 and 2)	NCT02465957, NCT03167164
GLA-SE	Toll-like receptor 4 agonist	Phase 1 (Completed)	NCT02035657
Tavokinogene telseplasmid	IL-12 plasmid (CD8+ T -cell and NK cell stimulation)	Phase 2 (Completed)	NCT01440816
F16IL2	F16-IL2 fusion protein for NK cell stimulation	Phase 2 (Unknown status)	NCT02054884
Talimogene Laherparepvec (TVEC)	Oncolytic HSV-1 encoding GM-CSF	Multiple phase 2 (Recruiting)	NCT02819843, NCT02978625

ABBV-368	Immunotherapy (not further specified)	Phase 1 (Recruiting)	NCT03071757
ETBX-051	Vaccine (not further specified)	Phase 1/2 (Not yet recruiting)	NCT03167164
ETBX-061	Vaccine (not further specified)	Phase 1/2 (Not yet recruiting)	NCT03167164
**mTOR inhibitors**			
Temsirolimus	mTOR inhibitor	Phase 1 (Completed)	NCT01155258
MLN0128	Dual inhibitor of raptor-mTOR and rictor-mTOR	Phase 1/2 (Recruiting)	NCT02514824
Everolimus	mTOR inhibitor	Multiple phase 1 (Active and completed)	NCT00655655, NCT01204476
**Somatostatin analogs**			
Octreotide Acetate	Somatostatin analog	Phase 1 (Completed)	NCT01204476
Pasireotide	Somatostatin analog	Phase 1 (Completed)	NCT01652547
Lanreotide	Somatostatin analog	Phase 2 (Completed)	NCT02351128
PEN-221	Somatostatin analog	Phase 1/2 (Recruiting)	NCT02936323
Bevacizumab	Anti-VEGF	Phase 1/2 (Not yet recruiting)	NCT03167164
**Receptor tyrosine kinase inhibitors**			
Imatinib mesylate	KIT inhibitor	Phase 2 (Completed)	NCT00068783
Cixutumumab	Anti-IGF1R antibody	Phase 1 (Completed)	NCT01204476
Cabozantinib	Inhibits multiple receptor tyrosine kinases	Phase 2 (Active)	NCT02036476
**Other targeted therapies**			
Oblimersen sodium	BCL2 antisense oligonucleotide	Phase 2 (Completed)	NCT00079131
BB-10901	Anti-CD56 antibody conjugated to microtubule assembly inhibitor	Phase 1 (Completed)	NCT00346385
**Conventional chemotherapeutics**			
Cyclophosphamide, fluorouracil, methotrexate		Phase 2 (Completed)	NCT00003549
Irinotecan hydrochloride		Phase 2 (Completed)	NCT00004922
Capecitabine, temozolomide		Phase 2 (Completed)	NCT00869050
Carboplatin, etoposide		Phase 2 (Active)	NCT01013779

Supplementary Box 1. Working Group Members
International Workshop on Merkel cell carcinoma Research, March 5-6, 2018

Name and Degree	Affiliation
Jürgen C. Becker, MD, PhD	German Cancer Consortium /DKTK - Partner site Essen/Düsseldorf; DKFZ, Im Neuenheimer Feld 280, 69120 Heidelberg, Germany; and Translational Skin Cancer Research; Dermatology, University Hospital Essen, Hufeland Strasse 55, 45147 Essen, Germany
John A. Beutler, PhD	Molecular Targets Program, Center for Cancer Research, National Cancer Institute, Bldg 576 Rm 103, NCI at Frederick, Frederick, MD 21702
Shailender Bhatia, MD	Medical Oncology, University of Washington/Fred Hutchinson Cancer Research Center, 825 Eastlake Ave E., Mailstop # CE2-102, Seattle, WA 98109
Christopher Bichakjian, MD	Division of Cutaneous Surgery and Oncology, Michigan Medicine - University of Michigan, 1500 E Medical Center Dr, UH South Rm 7680, Ann Arbor, MI 48109-5218
Isaac Brownell, MD, PhD	Dermatology Branch, National Institute of Arthritis and Musculoskeletal and Skin Diseases and Center for Cancer Research, National Cancer Institute, 10 Center Drive 12N240C, Bethesda, MD 20892-1908
Christopher B. Buck, PhD	Laboratory of Cellular Oncology, NCI, NIH, Building 37, Room 4118, Bethesda, MD 20892
Jaehyuk Choi, MD, PhD	Departments of Dermatology and Department of Biochemistry and Molecular Genetics, Northwestern University Feinberg School of Medicine, 303 East Superior St., Chicago, IL 60611
Daniel Coit, MD, FACS	Department of Surgery, Memorial Sloan Kettering Cancer Center
Sandra P. D'Angelo, MD	Department of Medicine Memorial Sloan Kettering Cancer Center and Weill Cornell Medical College
James A. DeCaprio, MD	Dana-Farber Cancer Institute, Department of Medical Oncology, Harvard Medical School, Department of Medicine, 450 Brookline Avenue; Mayer 440; Boston MA, 02215
Andrzej A. Dlugosz, MD	Department of Dermatology, University of Michigan, 1500 E. Medical Center Dr., 3431 Rogel Cancer Center, Ann Arbor, MI, 48109 SPC5932, USA
Elena Ezhkova, PhD	Black Family Stem Cell Institute, Department of Cell, Developmental, and Regenerative Biology; Icahn School of Medicine at Mount Sinai; 1 Gustave L. Levy Place, New York, NY 10029; USA
Nicole Fischer, PhD	Institute for Medical Microbiology, Virology and Hygiene, University Medical Center Hamburg-Eppendorf, Martinistrasse 46, 20246 Hamburg, Germany
Bernard A. Fox, PhD	Earle A Chiles Research Institute; 4805 NE Glisan, 2N56 North Pavilion, Portland, OR 97213
Denise A. Galloway, PhD	Division of Human Biology, Fred Hutchinson Cancer Research Center, 1100 Fairview Ave. N. Seattle, WA 98109
Ling Gao, MD, PhD	Department of Dermatology, UC-Irvine; and Dermatology Section, The Tibor Rubin VA Medical Center, 5901 East 7th Street, Long Beach, CA 90822
Brian R. Gastman, MD	Dermatology and Plastic Surgery Institute, Department of Plastic Surgery; Taussig Cancer Center; Lerner Research Institute; Department of Immunology; and Head and Neck Institute, Cleveland Clinic, 9500 Euclid Ave, Desk A60, Cleveland OH 44195
Sine Reker Hadrup, PhD	Technical University of Denmark, Kemitorvet, Building 204, Room 154, 2800 Kgs. Lyngby, Denmark
Matthew Hall, PhD	NCATS Chemical Genomics Center, National Center for Advancing Translational Sciences, National Institutes of Health, 9800 Medical Center Drive, Rockville, MD 20850, United States
Kelly Harms, MD, PhD	Division of Cutaneous Surgery and Oncology, Michigan Medicine - University of Michigan, 1500 E Medical Center Dr, UH South Rm 7680, Ann Arbor, MI 48109-5218
Paul W. Harms, MD, PhD	Departments of Pathology and Dermatology, 3261 Medical Science I, 1301 Catherine St, Ann Arbor, Michigan 48109-5602
Meliessa Hennessy, MPH	Global Clinical Development, Immuno-Oncology; EMD Serono Research & Development Institute, Inc; 45A Middlesex Turnpike; Billerica, MA 01821
Jennifer Kuhns, PhD	EMD Serono, Inc., One Technology Place, Rockland, MA 02370

Ellen A. Lumpkin, PhD	Dept. of Physiology & Cellular Biophysics, Columbia University College of Physicians & Surgeons, 1150 St Nicholas Ave Room 302N, New York NY 10032 USA
Jane L. Messina, MD	Departments of Anatomic Pathology and Cutaneous Oncology, Moffitt Cancer Center, 12902 Magnolia Drive Tampa, FL 33612
David M. Miller, MD, PhD	Department of Medicine, Division of Hematology/Oncology, Beth Israel Deaconess Medical Center, 330 Brookline Ave, Boston MA, 02215
Meagan Montesion, PhD	Foundation Medicine, Inc., 150 Second Street, Cambridge, MA, 02141, USA
Patrick S. Moore, MD, MPH	Department of Microbiology and Molecular Genetics, University of Pittsburgh, 1.8C Hillman Cancer Center, 5117 Centre Avenue, Pittsburgh, PA 15213-1862
Paul Nghiem, MD, PhD	Fred Hutchinson Cancer Research Center, 1100 Fairview Ave. N., P.O. Box 19024, Seattle, WA 98109-1024
Michael Oberst, PhD	Medimmune, One MedImmune Way, Gaithersburg, MD 20878
Kelly G. Paulson, MD, PhD	University of Washington/Fred Hutchinson Cancer Research Center, 1100 Fairview Ave N, Seattle, WA 98109
Guilherme Rabinowits, MD	Miami Cancer Institute, 8900 N. Kendall Dr., Miami, FL 33176
Betsy Read-Connole PhD	Cancer Etiology, CIHEB, Division of Cancer Biology, NCI, NIH, 9609 Medical Center Drive Room 6W534, Rockville, MD 20850-9748 USA
Karlyne M. Reilly, PhD	Rare Tumor Initiative, Pediatric Oncology Branch, Center for Cancer Research, NCI, 37 Convent Dr, Rm 1056, Bethesda, MD, 20814
Merrick Ross, MD	Department of Surgical Oncology, University of Texas MD Anderson Cancer Center, 1400 Pressler Street - Unit # 1484, Houston, Texas, 77030
Masahiro Shuda, PhD	Dept. of Microbiology and Molecular Genetics, University of Pittsburgh, 5117 Centre Ave, Pittsburgh, PA 15213
Arthur Sober, MD	Department of Dermatology, Massachusetts General Hospital, Bartlett 622, 40 Blossom Street, Boston, Massachusetts 02114
Ibrahima Soumaoro, MD	Immuno-Oncology Clinical Development, Bristol-Myers Squibb Company, 3401 Princeton Pike, Lawrenceville, NJ 08648
Gabriel J. Starrett, PhD	Laboratory of Cellular Oncology, CCR, NCI, NIH. Building 37, Room 4124, Bethesda, MD 20892
Michael Tetzlaff, MD, PhD	Departments of Pathology and Translational and Molecular Pathology, UT MD Anderson Cancer Center 1515 Holcomb Blcd, Unit 85, Houston, Texas 77030
Manisha Thakuria, MD	Department of Dermatology, Dana-Farber Cancer Institute/Brigham and Women's Hospital, Harvard Medical School, Boston, MA USA
Giovanna Tosato, MD	Laboratory of Cellular Oncology, NCI, NIH, Building 37, Room 4124, Bethesda, MD 20892
Richard Tothil, PhD	Centre for Cancer Research and Department of Clinical Pathology, University of Melbourne, 305 Grattan Street, Melbourne, Australia, 3000
Danielle Townsley, MD	MedImmune, Gaithersburg, MD
Connie Liu Trimble, MD	Departments of Gynecology and Obstetrics, Oncology, and Pathology, The Johns Hopkins Medical Institutions, Phipps 255, 600 North Wolfe St, Baltimore, MD 21287
Kenneth Y. Tsai, MD, PhD	Departments of Anatomic Pathology and Cutaneous Oncology, Moffitt Cancer Center, 12902 Magnolia Drive Tampa, FL 33612
Monique E. Verhaegen, PhD	Department of Dermatology, University of Michigan, 1500 E. Medical Center Dr., 3431 Rogel Cancer Center, Ann Arbor, MI, 48109 SPC5932, USA
Michael K. K. Wong, MD, PhD	UT MD Anderson Cancer Center, 1400 Holcombe Blvd., Unit 0430, Houston, TX 77030
Cassian Yee, MD	Department of Melanoma Medical Oncology, UT MD Anderson Cancer Center
Jianxin You, PhD	Department of Microbiology, University of Pennsylvania Perelman School of Medicine, 3610 Hamilton Walk, Philadelphia, PA 19104-6076, USA
Siegrid S. Yu, MD	Department of Dermatology, University of California, San Francisco Department of Dermatology, 1701 Divisadero Street, Third Floor San Francisco, CA 94115
Axel zur Hausen, MD, PhD	Department of Pathology and GROW-School for Oncology and Developmental Biology, Maastricht University Medical Center, Department of Pathology, P. Debyelaan 25, 6229 HX Maastricht, The Netherlands

Supplementary Box 2. Extended List of IWMCC Research Questions

Basic Science Questions

1. What is the cell of origin for MCC?
2. How does the MCC tumor microenvironment contribute to pathogenesis and immune evasion?
3. What are the relative contributions of ST and LT to tumor initiation and maintenance?
4. How do we model VP-MCC in mice, including features such as immune response/evasion, invasion, and metastasis?
5. What cell types harbor productive MCPyV infection?
6. Do VN-MCC (high UV damage burden) and VP-MCC (low UV damage burden) arise from distinct cell populations?
7. Do LT and ST affect the immune response?
8. How do we define viral latency, and what host and viral factors determine MCPyV latency?
9. How do non-MCPyV polyomaviruses contribute to human disease, especially tumorigenesis?
10. How do oncogene activation events (e.g. MYCL amplification) influence behavior of VN-MCC tumors?
11. What are the oncogenic drivers in VN-MCC tumors that lack RAS, PI3K, or MYCL activation?
12. How does normal Merkel cell biology inform MCC biology?
13. How do the functional regions of ST and LT contribute to their biology?
14. Does the type of immune suppression modulate viral replication?
15. What are the mechanisms of viral integration and LT mutation?
16. Are other cancers promoted by MCPyV?
17. What host and viral factors determine viral replication?
18. What genetic alterations can produce a mouse model of VN-MCC?
19. Why do transgenic MCPyV oncoproteins not readily cause tumors in rodents?
20. What determines splicing regulation of ST and LT?
21. How can we make animal models for VP and VN-MCC?
22. What are the appropriate models for MCC beyond mouse?
23. What is the role of MCPV for initiation or progression of MCC?
24. Are there structural differences between MCPyV T Antigens and corresponding SV40 proteins that are informative to the biology of these viruses?

Translational Questions

1. What are the potential therapeutic targets in VP-MCC?
2. What oncogenic drivers can be targeted to improve outcomes in MCC?
3. Do genomic alterations—either global (e.g. mutation burden) or specific (e.g. PIK3CA mutation)—have implications for prognosis or therapeutic response?
4. What are the potential therapeutic targets in VN-MCC?
5. What is the optimal detection method for MCPyV?
6. What is the value of distinguishing VP-MCC and VN-MCC?
7. Can LT and ST be targeted in VP-MCC?
8. What prognostic factors are most useful in predicting MCC disease course?
9. If most tumors are VP-MCC and have no UV-signature mutations, why does MCC have a predilection for sun-exposed skin and fair-skinned individuals?
10. How will routine use of immunotherapy alter the natural history and prognosis of MCC?
11. Are there additional therapeutic targets in VP-MCC and VN-MCC?
12. Is there a clinical need for MCPyV testing at diagnosis?

13. If MCPyV infection is common in most age groups, why is MCC primarily in the elderly and immunocompromised?
14. Do known risk factors for MCC (e.g. immune suppression, CLL, transplant, age, fair skin) differentially impact the risk for VP-MCC versus VN-MCC?
15. What is the relationship of other coincident cancers (e.g. CLL) to MCC?
16. Are the excess cases of MCC in immunocompromised populations due primarily to MCPyV?
17. How does tumor MCPyV status impact biomarker interpretation?
18. What is the potential for therapeutic antiviral therapy that can target MCPyV oncoproteins?
19. What is the utility of ST antigen serology/other biomarkers in clinical follow-up, prognosis?
20. When should clinical management be altered based on prognostic markers?
21. How reliable are immune-based markers in immunosuppressed patients?
22. What factors account for the changing incidence of MCC over time and by geographical region?
23. Does UV exposure account for all the excess of VN-MCC observed in Australia?
24. What are the pathways that drive MCC progression?
25. What is the best approach to implementing PDX models?
26. What are the rejection antigens targeted in immunotherapy?
27. What markers most reliably distinguish MCC from metastatic non-cutaneous small cell carcinoma (especially for CK20-negative MCC)?

Clinical Questions

1. What alternatives to immunotherapy are effective for nonresponders and ineligible patients?
2. What markers or tumor characteristics predict response to immune checkpoint inhibitors?
3. What is the optimal role for radiotherapy in MCC management?
4. What treatment combinations will enhance the efficacy of immunotherapy for MCC?
5. How can clinical trials be optimized to maximize collaboration and minimize competition for this rare tumor?
6. Is there benefit to adjuvant use of immunotherapy for MCC?
7. Is combinatorial use of targeted therapy and immunotherapy effective?
8. Would multi-institutional synoptic reporting of clinical, pathology, and genetic data from MCC patients to a registry be useful and feasible?
9. What is the optimal surgical management of MCC?
10. What is the optimal management of clinically occult nodal disease?
11. How can clinical observations from centers caring for MCC patients be shared to help instruct basic and translational research?
12. What is the optimal management of MCC considering therapeutic risks and benefits?
13. How does MCC management impact the patient and how can patient experiences be improved?
14. Does lymph node dissection reduce the efficacy of immunotherapy in MCC?
15. Will FDA approval of avelumab for advanced MCC require head-to-head comparison trials of other immunotherapies?
16. What is the best approach to facilitating communication and collaboration via a listserv (ITSCC)?
17. What are useful predictive and prognostic biomarkers? What biomarkers are informative in guiding treatment decisions?
18. What are mechanisms of escape from checkpoint blockade (PD-(L)1)?
19. What hypothesis-driven research questions might benefit from a multicenter data registry?
